# TNF-induced necroptosis initiates early autophagy events via RIPK3-dependent AMPK activation, but inhibits late autophagy

**DOI:** 10.1080/15548627.2021.1899667

**Published:** 2021-03-28

**Authors:** Wenxian Wu, Xiaojing Wang, Yadong Sun, Niklas Berleth, Jana Deitersen, David Schlütermann, Fabian Stuhldreier, Nora Wallot-Hieke, María José Mendiburo, Jan Cox, Christoph Peter, Ann Kathrin Bergmann, Björn Stork

**Affiliations:** a Institute of Molecular Medicine I, Medical Faculty, Heinrich Heine University Düsseldorf, Düsseldorf, Germany; b DWI-Leibniz Institute for Interactive Materials, Aachen, Germany; c Core Facility for Electron Microscopy, Medical Faculty, Heinrich Heine University Düsseldorf, Düsseldorf, Germany

**Keywords:** AMPK, autophagy, necroptosis, RIPK3, STX17

## Abstract

Macroautophagy/autophagy and necroptosis represent two opposing cellular s tress responses. Whereas autophagy primarily fulfills a cyto-protective function, necroptosis is a form of regulated cell death induced via death receptors. Here, we aimed at investigating the molecular crosstalk between these two pathways. We observed that RIPK3 directly associates with AMPK and phosphorylates its catalytic subunit PRKAA1/2 at T183/T172. Activated AMPK then phosphorylates the autophagy-regulating proteins ULK1 and BECN1. However, the lysosomal degradation of autophagosomes is blocked by TNF-induced necroptosis. Specifically, we observed dysregulated SNARE complexes upon TNF treatment; e.g., reduced levels of full-length STX17. In summary, we identified RIPK3 as an AMPK-activating kinase and thus a direct link between autophagy- and necroptosis-regulating kinases.

**Abbreviations:** ACACA/ACC: acetyl-CoA carboxylase alpha; AMPK: AMP-activated protein kinase; ATG: autophagy-related; BECN1: beclin 1; GFP: green fluorescent protein; EBSS: Earle’s balanced salt solution; Hs: *Homo sapiens*; KO: knockout; MAP1LC3/LC3: microtubule associated protein 1 light chain 3; MEF: mouse embryonic fibroblast; MLKL: mixed lineage kinase domain like pseudokinase; Mm: *Mus musculus*; MTOR: mechanistic target of rapamycin kinase; MVB: multivesicular body; PIK3C3/VPS34: phosphatidylinositol 3-kinase catalytic subunit type 3; PIK3R4/VPS15: phosphoinositide-3-kinase regulatory subunit 4; PLA: proximity ligation assay; PRKAA1: protein kinase AMP-activated catalytic subunit alpha 1; PRKAA2: protein kinase AMP-activated catalytic subunit alpha 2; PRKAB2: protein kinase AMP-activated non-catalytic subunit beta 2; PRKAG1: protein kinase AMP-activated non-catalytic subunit gamma 1; PtdIns3K: phosphatidylinositol 3-kinase; PtdIns3P: phosphatidylinositol-3-phosphate; RIPK1: receptor interacting serine/threonine kinase 1; RIPK3: receptor interacting serine/threonine kinase 3; SNAP29: synaptosome associated protein 29; SNARE: soluble N-ethylmaleimide-sensitive factor attachment protein receptor; SQSTM1/p62: sequestosome 1; STK11/LKB1: serine/threonine kinase 11; STX7: syntaxin 7; STX17: syntaxin 17; TAX1BP1: Tax1 binding protein 1; TNF: tumor necrosis factor; ULK1: unc-51 like autophagy activating kinase 1; VAMP8: vesicle associated membrane protein 8; WT: wild-type.

## Introduction

(Macro-)Autophagy is an intracellular recycling process that is characterized by vesicle-mediated transfer of cargo to lysosomes. Several stimuli are able to induce autophagy, e.g. nutrient starvation, hypoxia, protein aggregation, or infection with intracellular pathogens [1]. Upon energy or nutrient deprivation, the energy-sensing AMP-activated protein kinase (AMPK) can be activated by STK11/LKB1 (serine/threonine kinase 11) [2–4]. Subsequently, activated AMPK can in turn regulate downstream substrates via phosphorylation, e.g. the autophagy-inducing ULK1 (unc-51 like autophagy activating kinase 1) or MTOR (mechanistic target of rapamycin kinase) [5,6]. The inhibition of MTOR by AMPK can be mediated indirectly by phosphorylating TSC2 (TSC complex subunit 2) at S1387 (human amino acid sequence) or/and directly by phosphorylating regulatory associated protein of MTOR complex 1 at S722 and S792 (human and murine amino acid sequence) [7]. MTOR inhibition then results in the dissociation of MTOR from the ULK1 complex, in the dephosphorylation of MTOR-dependent phospho-sites in ULK1 (S638 and S758, human amino acid sequence), and in the activation of ULK1 [5,8]. AMPK can also directly phosphorylate and thus regulate ULK1. AMPK-dependent phospho-sites in ULK1 include S317, S556, and S638 (human amino acid sequence) [8–12]. The active ULK1 complex then translocates to specific subdomains of the ER in order to initiate the nucleation of autophagosomes [1]. To date, several subdomains of the ER have been proposed as “hot spots” for autophagosome formation [13]. Subsequently, the class III phosphatidylinositol 3-kinase (PtdIns3K) is recruited to these subdomains. This lipid kinase complex is composed of the catalytic subunit PIK3C3/VPS34 (phosphatidylinositol 3-kinase catalytic subunit type 3), and the associated proteins BECN1 (beclin 1), PIK3R4/VPS15 (phosphoinositide 3-kinase regulatory subunit 4), ATG14 and nuclear receptor binding factor 2 [14]. The class III PtdIns3K complex is also regulated by ULK1- and AMPK-dependent phosphorylations of several subunits [15–19]. Activated PtdIns3K produces phosphatidylinositol-3-phosphate (PtdIns3P), leading to the formation of membranous platforms termed omegasomes/ER cradles that contain the PtdIns3P-binding protein ZFYVE1/DFCP1 (zinc finger FYVE-type containing 1) and that give rise to phagophores [20–23]. This process is supported by the recruitment of further autophagy-specific components, e.g. the PtdIns3P-binding protein WD repeat domain, phosphoinositide interacting 2 or components of the two autophagy-specific ubiquitin-like conjugation machineries [1].

In addition to STK11/LKB1-mediated AMPK activation, both CAMKK2 (calcium/calmodulin dependent protein kinase kinase 2) and MAP3K7 (mitogen-activated protein kinase kinase kinase 7) activate AMPK, depending on the cytosolic Ca^2+^ level or cytokine treatment, respectively [24–27]. AMPK is composed of a catalytic α-subunit (PRKAA1/AMPKα1 or PRKAA2/AMPKα2), a scaffold β-subunit (PRKAB1/AMPKβ1 or PRKAB2/AMPKβ2) and a regulatory γ-subunit (PRKAG1/AMPKγ1, PRKAG2/AMPKγ2 or PRKAG3/AMPKγ3) [28]. All three AMPK-activating kinases phosphorylate the α1-subunit PRKAA1 in the activation loop at T183, which corresponds to T172 in PRKAA2 [28]. Interestingly, Dalle Pezze et al. recently reported that AMPK is not only activated under stress or starvation conditions, but can also positively regulate autophagy under amino acid sufficiency [29].

In contrast to the cytoprotective function of autophagy, necroptosis represents an inflammatory form of regulated cell death and is characterized by a necrotic morphotype [30]. Although necroptosis can be triggered by several stimuli, induction mediated by death receptors (e.g., TNFRSF1A/TNFR1 [TNF receptor superfamily member 1A]) is the best characterized form [31,32]. Auto- and trans-phosphorylation of RIPK1 (receptor-interacting serine/threonine kinase 1) and RIPK3 (receptor interacting serine/threonine kinase 3) induce the formation of an amyloid-like multiprotein complex termed necrosome. Necroptosis and the assembly of necrosomes can be inhibited by CASP8 (caspase 8)-dependent cleavage of RIPK1 or RIPK3 [33–35]. When CASP8 is inhibited by pharmacological compounds (e.g., Q-VD-OPh or z-VAD-FMK) or under certain physiological conditions (e.g. viral infections), necroptosis is initiated [36–38]. Activated RIPK3 recruits and activates MLKL (mixed lineage kinase domain like pseudokinase), which in turn translocates to the plasma membrane and mediates necrotic membrane disruption [31,32,39].

Both autophagy and necroptosis balance cell death and survival. Although there are some reports indicating a crosstalk between these two stress responses, the mechanistic details remain poorly understood so far. Here, we show that TNF-induced necroptosis blocks the lysosomal degradation of autophagosomes, presumably via the dysregulation of the autophagosome-lysosome fusion-mediating SNARE (soluble N-ethylmaleimide-sensitive factor attachment protein receptor) proteins. In detail, we observe reduced interactions of VAMP8 (vesicle associated membrane protein 8), STX17 (syntaxin 17) and STX7 (syntaxin 7) with SNAP29 (synaptosome associated protein 29), and a decrease of full-length STX17. In contrast, it appears that early autophagy signaling events such as ULK1 and BECN1 phosphorylation are induced under pro-necroptotic conditions and in a RIPK3-dependent manner. We observe that RIPK3 interacts with AMPK and that RIPK3 phosphorylates the catalytic subunit PRKAA1 at T183 in order to activate AMPK. Subsequently, pro-autophagic AMPK substrates such as ULK1 and BECN1 become phosphorylated. Collectively, we have identified RIPK3 as AMPK-activating kinase and thus a molecular crosstalk between the autophagy- and necroptosis-regulating kinases.

## Results

### TNF-induced necroptosis is accompanied by AMPK activation and phosphorylation of ULK1 and BECN1

**Figure 1.**
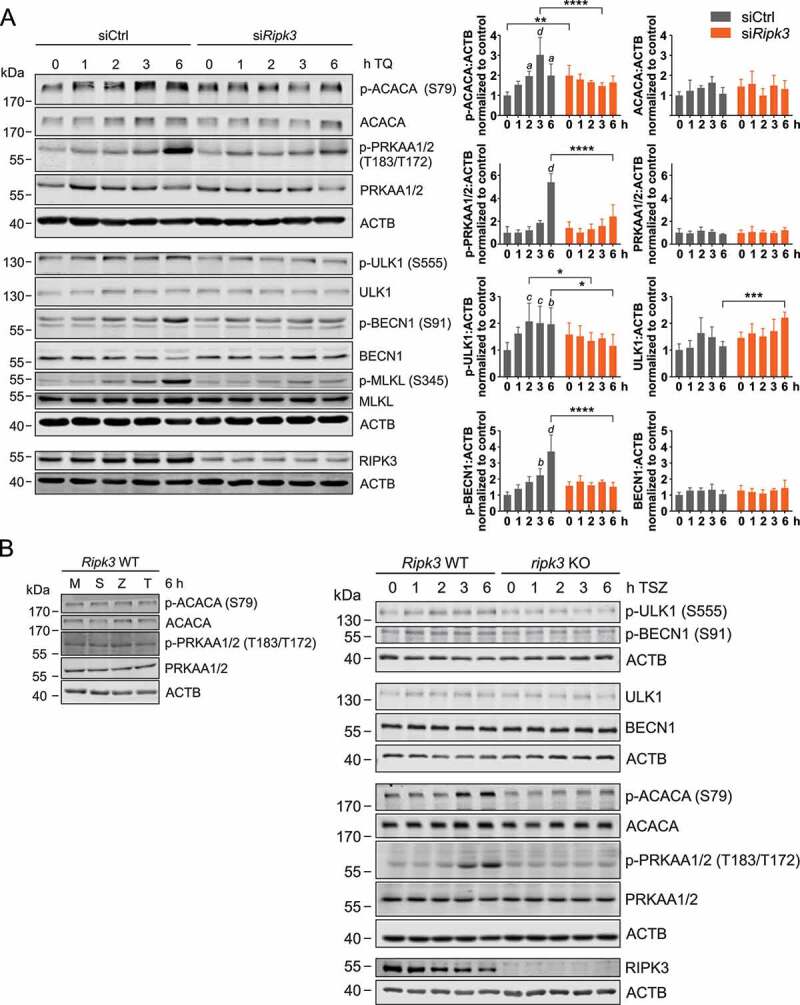
TNF treatment induces activation of AMPK. (**A**) L929 cells were transfected with non-targeting (siCtrl) or *Ripk3* siRNAs (si*Ripk3*). 48 h post transfection, cells were exposed to 10 ng/ml TNF and 30 µM QVD (TQ) for the indicated times. Then, whole cell lysates were subjected to SDS-PAGE and immunoblotting for indicated proteins. A compilation of representative immunoblots is shown; three ACTB immunblots are shown, but each protein was normalized to its corresponding loading control. The density of each protein band was divided by the average of the density of all bands from the same protein on the membrane. Fold changes were calculated by dividing each normalized ratio (protein to loading control) by the average of the ratios of the control lane (scr, 0 h TQ). Results are mean + SD from at least 3 independent experiments. Statistical analysis was done by repeated measures two-way ANOVA (corrected by Sidak’s multiple comparisons test between siRNAs and corrected by Tukey’s multiple comparisons test between time points). Statistically significant differences within non-targeting siRNA-transfected cells (compared to scr, 0 h TQ) are depicted as letters directly above the bars. * or *a: P* < 0.05, ** or *b: P* < 0.01, *** or *c: P* < 0.001, **** or *d: P* < 0.0001. (**B**) *Ripk3* WT and KO MEFs were exposed to indicated treatments (medium [M], 30 ng/ml TNF [T], 100 nM SMAC-mimetic [S], 20 µM z-VAD [Z]) for indicated times. Then, cells were lysed and cleared cellular lysates were subjected to SDS-PAGE and analyzed by immunoblotting for indicated proteins

Treatment of murine L929 fibroblasts with TNF in combination with the caspase inhibitor QVD (hereafter abbreviated as TQ) leads to the induction of necroptosis (Figure S1A). In order to investigate the effect of necroptosis on autophagy, we initially monitored early autophagy signaling events under these pro-necroptotic conditions. We observed increased activity of AMPK, as detected by immunoblotting for phospho-PRKAA1 (T183), phospho-ACACA (S79), phospho-ULK1 (S555, murine sequence; corresponding to S556 in human ULK1), and phospho-BECN1 (S91, murine sequence; corresponding to S93 in human BECN1) (Figure 1A). These increased phosphorylation events were clearly dependent on RIPK3 signaling, since they were abolished by siRNA-mediated RIPK3 knockdown. Similar results were obtained when we used a RIPK3 inhibitor (GSK’872 [40]) instead of *Ripk3* siRNA (Figure S1B). In order to confirm these observations in an alternative cellular model system, we made use of wild-type (WT) and *ripk3* KO murine embryonic fibroblasts (MEFs). Here, we induced necroptosis by treatment with TNF in combination with a DIABLO/Smac mimetic (here: Birinapant) and the pan-caspase inhibitor zVAD (hereafter abbreviated as TSZ) as previously reported [37], and neither of these components alone affected AMPK activity (Figure 1B, left panels). TSZ induced AMPK activation and phosphorylation of the AMPK substrates ACACA, ULK1 and BECN1 in wild-type MEFs, whereas these processes were inhibited in *ripk3* KO MEFs (Figure 1B, right panels).

**Figure 2.**
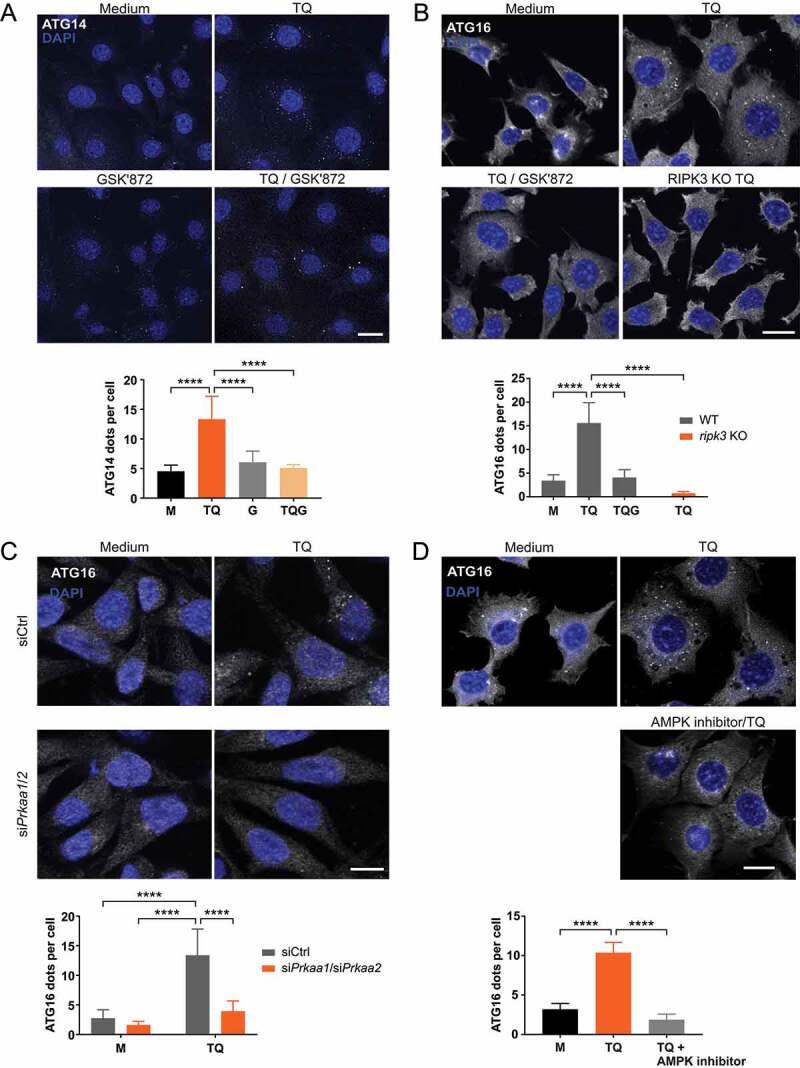
TNF treatment induces ATG14 and ATG16L1 puncta formation via RIPK3 and AMPK. (**A and B**) WT and *ripk3* KO L929 cells were exposed to indicated treatments (medium [M], 10 ng/ml TNF [T], 30 µM QVD [Q], 5 µM GSK’872 [G]) for 3 h. After that, cells were fixed and subjected to ATG14 (A) or ATG16L1 (B) immunostaining using anti-ATG14 (Santa Cruz Biotechnology, sc-164767) or anti-ATG16L1 antibodies (MBL International, PM040) and IRDye® 680RD donkey anti-goat or Alexa Fluor®488-conjugated goat anti-rabbit IgG (H + L) secondary antibodies. Puncta quantification was done using ImageJ software. Data represent mean + SD. A minimum of 120 (A) or 261 cells (B) was analyzed. (**C**) WT L929 cells were transfected with non-targeting (siCtrl) or *Prkaa1/Prkaa2* siRNAs (si*Prkaa1/*si*Prkaa2*). 48 h post transfection, cells were left untreated (medium, M) or exposed to 10 ng/ml TNF and 30 µM QVD (TQ) for 3 h. Then, cells were fixed and subjected to ATG16L1 immunostaining using anti-ATG16L1 antibodies (MBL International, PM040) and Alexa Fluor®488-conjugated goat anti-rabbit IgG (H + L) secondary antibodies. Puncta quantification was done using ImageJ software. Data represent mean + SD. A minimum of 655 cells was analyzed. (**D**) WT L929 cells were left untreated (medium, M) or exposed to indicated treatments (10 ng/ml TNF [T], 30 µM QVD [Q], 5 µM AMPK inhibitor dorsomorphin) for 3 h. Then, cells were fixed and subjected to ATG16L1 immunostaining using anti-ATG16L1 antibodies (MBL International, PM040) and Alexa Fluor®488-conjugated goat anti-rabbit IgG (H + L) secondary antibodies. Puncta quantification was done using ImageJ software. Data represent mean + SD. A minimum of 122 cells was analyzed. (**A-D**) Statistical analysis was performed using ordinary one-way ANOVA (corrected by Tukey’s multiple comparisons test) for A, B and D; or two-way ANOVA (corrected by Tukey’s multiple comparisons test) for C. For B, statistical analysis was additionally performed using unpaired *t* test with Welch’s correction (TQ treatment of WT vs. *ripk3* KO cells). ****P < 0.0001. Scale bar: 20 µm

Generally, AMPK-dependent phosphorylation of ULK1 at S555 (human S556) and of BECN1 at S91 (human S93) have been associated with the induction of autophagy [10,16,41,42]. Accordingly, we speculated that initial autophagy signaling events are activated upon TQ- or TSZ-induced necroptosis in a RIPK3-dependent manner. To test this hypothesis, we tested downstream markers of autophagy by immunofluorescence in L929 cells, e.g. ATG14 (Figure 2A) and ATG16L1 (Figure 2B). We observed that TQ treatment induces ATG14 puncta formation, and that this effect was significantly blocked by GSK’872-mediated inhibition of RIPK3 (Figure 2A). Similarly, TQ-induced ATG16L1 puncta formation was inhibited in *ripk3* KO L929 cells or in WT L929 cells treated with GSK’872 (Figure 2B). Of note, this ATG16L1 puncta formation was clearly dependent on AMPK, because both *Prkaa1/2* siRNA and treatment with the AMPK inhibitor dorsomorphin prevented TQ-induced ATG16L1 puncta formation (Figure 2C and Figure 2D). Taken together, it appears that TQ or TSZ treatment induces early autophagy signaling events via a RIPK3-AMPK signaling axis. We also investigated whether RIPK3 is also involved in canonical starvation-induced autophagy, but we did not observe a significant difference of EBSS-induced AMPK activation or LC3 lipidation between WT and *ripk3* KO L929 cells (Figure S2A). The RIPK3-dependent induction of early autophagy signaling events upon TQ or TSZ treatment might pursue a cyto-protective function and thus slow down the execution of necroptosis. Along these lines, necroptosis-inhibitory functions have been attributed to AMPK and to ULK1 [43–45]. Accordingly, we observed increased TQ-induced cell death in L929 cells upon PRKAA1/2 knockdown (Figure S2B) and increased TSZ-induced cell death in *prkaa1 prkaa2* or *ulk1 ulk2* double-knockout MEFs compared to wild-type control cells (Figures S2C and S2D).

### RIPK3 interacts with AMPK

**Figure 3.**
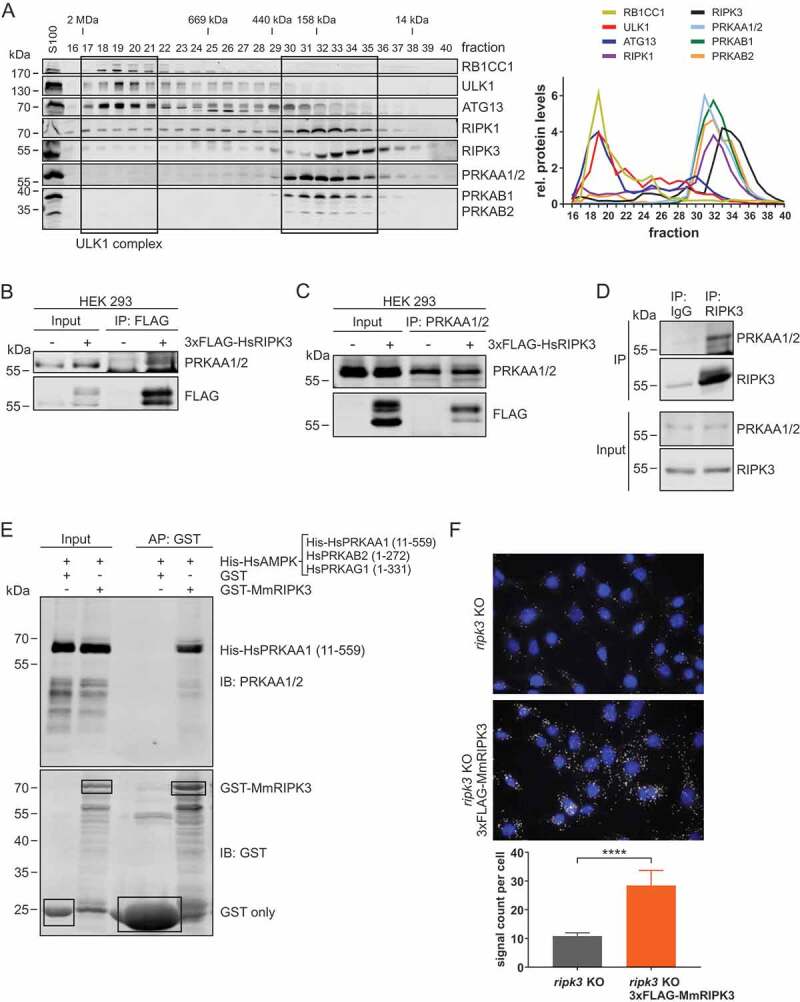
RIPK3 interacts with AMPK. (**A**) S100 extracts of MEFs were separated by size-exclusion chromatography on a Superose 6 increase column. Fractions were analyzed by immunoblotting for the indicated proteins. The diagram shows protein levels for fractions 16–40 and the density of each protein band was divided by the average of the density of all bands from the same protein on the membrane. (**B**) HEK293 cells were left untransfected or were transfected with a vector encoding 3xFLAG-HsRIPK3 for 24 h. Then, cells were lysed and cleared cellular lysates were subjected to immunopurification using anti-FLAG beads. Purified proteins were subjected to SDS-PAGE and analyzed by immunoblotting for FLAG and AMPK. (**C**) HEK293 cells were left untransfected or were transfected with a vector encoding 3xFLAG-HsRIPK3 for 24 h. Then, cells were lysed and cleared cellular lysates were subjected to immunopurification using anti-AMPK antibodies. Purified proteins were subjected to SDS-PAGE and analyzed by immunoblotting for FLAG and AMPK. (**D**) L929 cells were lysed and cleared cellular lysates were subjected to immunopurification using anti-IgG or anti-RIPK3 antibodies. Purified proteins were subjected to SDS-PAGE and analyzed by immunoblotting for AMPK and RIPK3. (**E**) GST or GST-MmRIPK3 immobilized on glutathione-Sepharose beads was incubated with His-AMPK [His-HsPRKAA1 (11–559) + HsPRKAB2 (1–272) + HsPRKAG1 (1–331)] overnight. After washing the beads, bound proteins were eluted by boiling for 10 min at 95°C. Proteins were subjected to SDS-PAGE and analyzed by immunoblotting for AMPK and GST. (**F**) *ripk3* KO MEFs were retrovirally transfected with empty vector or cDNA encoding 3xFLAG-MmRIPK3. Cells were seeded onto glass coverslips. The next day, cells were fixed and analyzed using proximity ligation assay as described in the material and methods section (anti-RIPK3: Prosci, 2283; anti-PRKAA1/2: Cell Signaling Technology, 2793). Nuclei were stained with DAPI. Signals and nuclei per image were counted and the signal:nuclei ratio was calculated. Data represent mean + SD. A minimum of 216 cells was analyzed. Statistical analysis was performed using an unpaired *t* test with Welch’s correction. ****P < 0.0001. Scale bar: 20 µm

Since we observed that the induction of necroptosis affected AMPK-dependent signaling and early autophagy events, we next investigated the crosstalk between AMPK and the pro-necroptotic RIPK3. In a first approach, we performed size exclusion chromatography of S100 lysates derived from MEFs (Figure 3A). We detected the ULK1 complex in high molecular mass fractions of approximately 3 MDa as previously described [46–48]. Of note, AMPK and both RIPK1 and RIPK3 were present in fractions corresponding to a lower molecular mass range of 14–158 kDa (Figure 3A). Since the presence of proteins in the same fractions does not prove a direct interaction, we next performed immunopurification experiments. Since we observed a RIPK3-dependent induction of early autophagy signaling events, we focused on RIPK3. We transiently transfected cDNA encoding 3xFLAG-HsRIPK3 into HEK293 cells. Following anti-FLAG immunopurification, we detected co-purification of endogenous AMPK (Figure 3B). In a similar approach, we co-purified 3xFLAG-HsRIPK3 with purified AMPK (Figure 3C). Furthermore, we also immunopurified endogenous AMPK with endogenous RIPK3 (Figure 3D). The direct interaction between RIPK3 and AMPK was also confirmed by affinity purification using recombinant proteins (Figure 3E). Finally, we confirmed the interaction between these two kinases on the cellular level by a proximity ligation assay (PLA). In this assay, single protein-protein interactions can be detected using antibody-recognition combined with exponential signal amplification by PCR. We transfected *ripk3* KO MEFs with cDNA encoding 3xFLAG-MmRIPK3. 3xFLAG-MmRIPK3 was stained with rabbit anti-RIPK3 antibodies and AMPK with mouse anti-PRKAA1/2 antibodies. As control, *ripk3* KO MEFs were transfected with empty vector. Cells reconstituted with 3xFLAG-MmRIPK3 displayed strong signals with significant difference to control cells (Figure 3F). Collectively, these data indicate that AMPK can associate with RIPK3.

### RIPK3 directly phosphorylates PRKAA1 at T183 and thus activates AMPK

**Figure 4.**
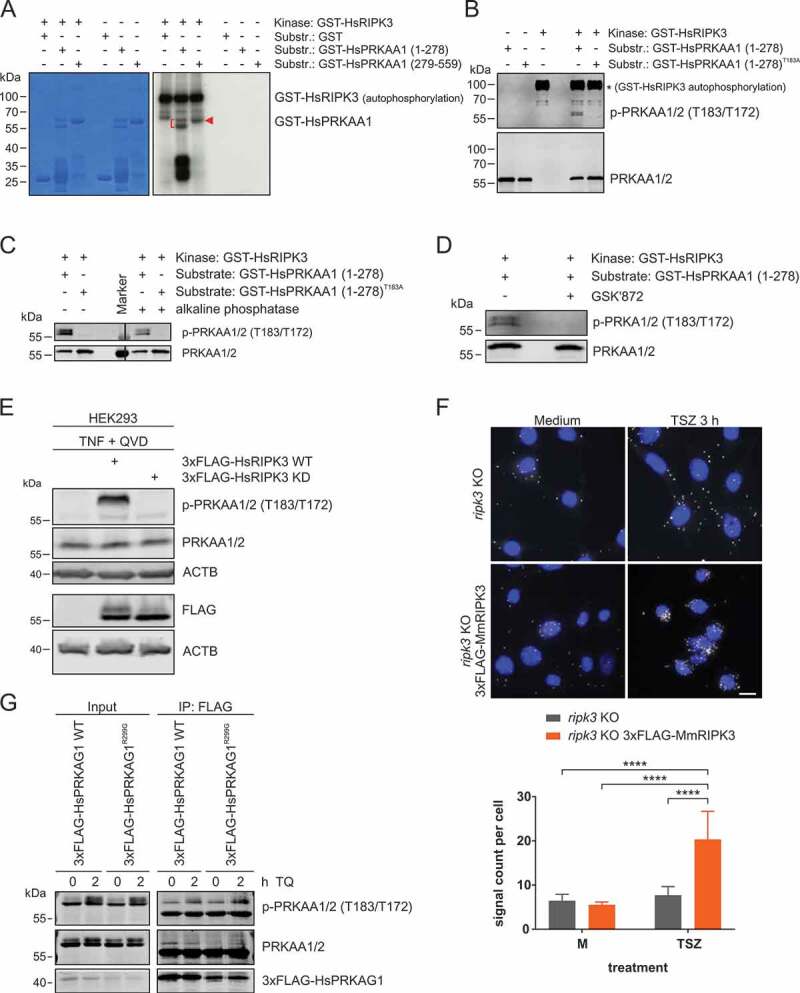
RIPK3 directly phosphorylates PRKAA1 at T183. (**A**) For *in vitro* kinase assay, purified GST, GST-HsPRKAA1(1–278) and GST-HsPRKAA1(279–559) were incubated with activated RIPK3 and [γ-^32^P]-ATP. The reactions were subjected to SDS-PAGE. After Coomassie Brilliant Blue staining and drying of the gels, autoradiography was performed. (**B**) GST-HsPRKAA1 WT and the T183A mutant were purified and were incubated with activated RIPK3 and cold ATP. The reactions were subjected to SDS-PAGE and analyzed by immunoblotting for phospho-PRKAA1/2 T183/T172 and AMPK. (**C**) GST-HsPRKAA1 WT and the T183A mutant were incubated with activated RIPK3 and cold ATP with or without alkaline phosphatase. The reactions were subjected to SDS-PAGE and analyzed by immunoblotting for phospho-PRKAA1/2 T183/T172 and AMPK. (**D**) GST-HsPRKAA1 WT was incubated with activated RIPK3 and cold ATP with or without 50 µM GSK’872. The reactions were subjected to SDS-PAGE and analyzed by immunoblotting for phospho-PRKAA1/2 T183/T172 and AMPK. (**E**) HEK293 cells were left untransfected or were transfected with cDNA encoding either 3xFLAG-HsRIPK3 WT or 3xFLAG-HsRIPK3 kinase-dead (KD) for 24 h. After that, cells were treated with 30 ng/ml TNF + 30 µM QVD for 24 h. Then, cells were lysed and cleared cellular lysates were subjected to SDS-PAGE and analyzed by immunoblotting for phospho-PRKAA1/2 T183/T172, AMPK, FLAG and ACTB. (**F**) *ripk3* KO MEFs were retrovirally transfected with empty vector or cDNA encoding 3xFLAG-MmRIPK3. Cells were seeded onto glass coverslips. The next day, the cells were left untreated (medium, M) or treated with 30 ng/ml TNF + 100 nM SMAC-mimetic + 20 µM z-VAD (TSZ) for 3 h. Then cells were fixed and analyzed using proximity ligation assay as described in the material and methods section (anti-phospho-PRKAA1/2 T183/T172: Cell Signaling Technology, 2535; anti-PRKAA1/2: Cell Signaling Technology, 2793). Nuclei were stained with DAPI. Signals and nuclei per image were counted and the signal:nuclei ratio was calculated. Data represent mean + SD. A minimum of 107 cells was analyzed. Statistical analysis was performed using ordinary two-way ANOVA (corrected by Tukey’s multiple comparisons test). ****P < 0.0001. Scale bar: 20 µm. (**G**) L929 cells were transiently transfected with cDNA encoding either 3xFLAG-HsPRKAG1 WT or R299G for 24 h. After that, cells were treated with or without 10 ng/ml TNF + 30 µM QVD (TQ) for 2 h. Then, cells were lysed and cleared cellular lysates were subjected to immunopurification using anti-FLAG beads. Purified proteins were subjected to SDS-PAGE and analyzed by immunoblotting for indicated proteins

Because we observed that RIPK3 interacts with AMPK, we next tested if RIPK3 can phosphorylate AMPK. In a first approach, we used recombinant RIPK3 as kinase and GST-HsPRKAA1 (1–278) or GST-HsPRKAA1 (279–559) as substrates in an *in vitro* kinase assay. We observed that RIPK3 can phosphorylate both truncated versions of PRKAA1 (Figure 4A). Since we observed that RIPK3 can regulate AMPK activity upon TQ or TSZ treatment (Figure 1A, Figures 1B and S1B), we wondered if RIPK3 could directly phosphorylate PRKAA1 at T183 to regulate AMPK activity. To test this hypothesis, we generated the mutant GST-HsPRKAA1 (1–278)^T183A^ and repeated the *in vitro* kinase assay with cold ATP. We performed immunoblotting for phospho-PRKAA1 T183 and observed a specific band for GST-HsPRKAA1 (1–278), but not for the GST-HsPRKAA1 (1–278)^T183A^ mutant (Figure 4B). This RIPK3-dependent phosphorylation of PRKAA1 at T183 was sensitive to both alkaline phosphatase and RIPK3 inhibitor GSK’872 treatment (Figure 4C and Figure 4D). To test if RIPK3 phosphorylates AMPK in intact cells, we treated HEK293 cells that express either 3xFLAG-HsRIPK3 WT or a kinase-dead (KD) variant with TQ for 24 h. We observed strong phospho-PRKAA1 T183 signal in HEK293 cells expressing 3xFLAG-HsRIPK3 compared to cells expressing kinase-dead 3xFLAG-HsRIPK3 (Figure 4E). Using again a proximity ligation assay, we also detected stronger PRKAA phosphorylation in *ripk3* KO MEFs reconstituted with 3xFLAG-MmRIPK3 compared to non-reconstituted *ripk3* KO MEFs (Figure 4F). Although these assays confirm the direct phosphorylation of AMPK by RIPK3, we wanted to investigate whether an additional mechanism contributes to AMPK activation during necroptosis. It has previously been described that the induction of necroptosis results in reduced cellular ATP levels [49,50]. In order to analyze the AMP-dependency of the observed AMPK activation, we expressed 3xFLAG-tagged wild-type PRKAG1 and an R299G variant in L929 cells. This mutation affects the binding of AMP at site 3 in human PRKAG1 [51]. Upon treatment with TQ and subsequent anti-FLAG immunopurification, we did not observe any differences in PRKAA1/2 T183/T172 phosphorylation between cells expressing wild-type or PRKAG1^R299G^ (Figure 4G). These data indicate that an intact AMP-binding site 3 on PRKAG is not required for TQ-induced AMPK activation. Altogether, our results indicate that RIPK3 phosphorylates PRKAA1 at T183 to regulate AMPK activity upon TNF-induced necroptosis. This RIPK3-dependent activation of AMPK then induces early autophagy events.

### TNF-induced necroptosis inhibits lysosomal autophagosome degradation

**Figure 5.**
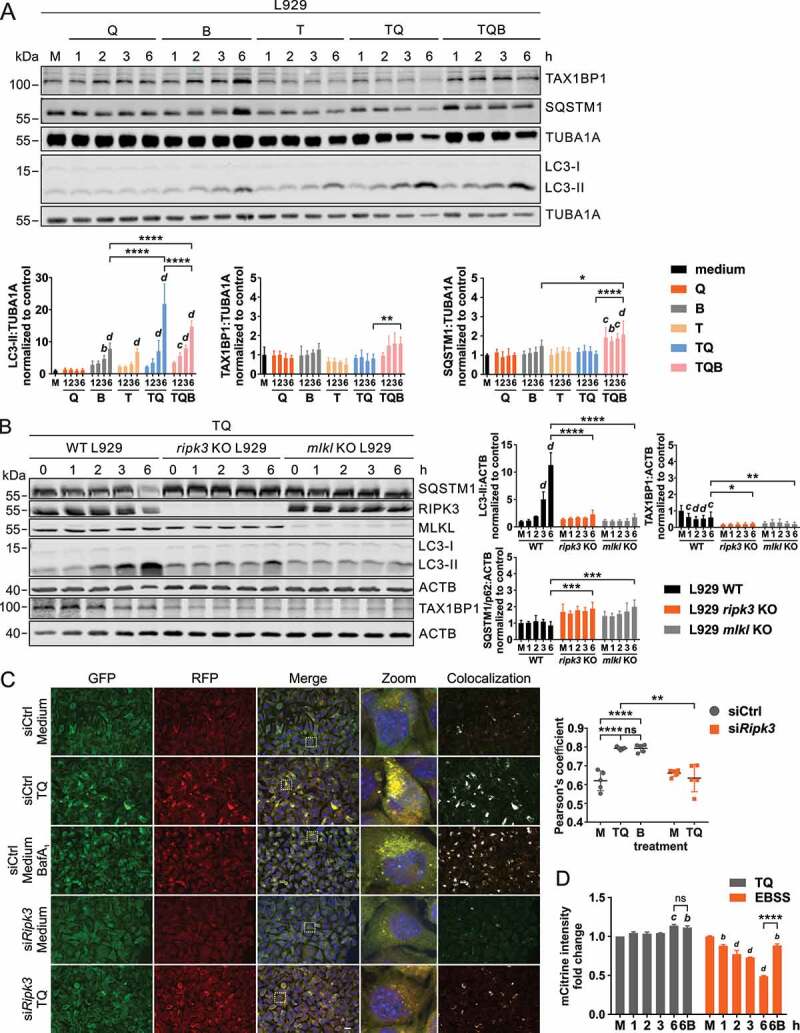
Necroptosis inhibits lysosomal LC3 degradation. (**A**) L929 cells were left untreated (medium, M) or exposed to 30 µM QVD (Q), 20 nM bafilomycin A_1_ (B), 10 ng/ml TNF (T), 10 ng/ml TNF + 30 µM QVD with or without 20 nM bafilomycin A_1_ (TQ or TQB) for indicated times. Then, cells were lysed and cleared cellular lysates were subjected to SDS-PAGE and immunoblotting for indicated proteins. The density of each protein band was divided by the average of the density of all bands from the same protein on the membrane. Fold changes were calculated by dividing each normalized ratio (protein to loading control) by the average of the ratios of the control lane (medium). Statistical graphics represents mean + SD (n = 4). (**B**) L929 WT, *ripk3* KO or MLKL KO cells were exposed to 10 ng/ml TNF and 30 µM QVD (TQ) for indicated times. Then, cells were lysed and cleared cellular lysates were subjected to SDS-PAGE and immunoblotting for indicated proteins. The density of each protein band was divided by the average of the density of all bands from the same protein on the membrane. Fold changes were calculated by dividing each normalized ratio (protein to loading control) by the average of the ratios of the control lane (medium). Statistical graphics represents mean + SD (n = 3). (**C**) L929 cells retrovirally transfected with cDNA encoding mRFP-EGFP-rLC3 were transfected with non-targeting (siCtrl) or *Ripk3* siRNAs (si*Ripk3*). 48 h post transfection, cells were left untreated (medium, M) or exposed to indicated treatments (10 ng/ml TNF [T], 30 µM QVD [Q], 20 nM bafilomycin A_1_ [B]) for 3 h. Then cells were fixed and RFP and GFP fluorescence was analyzed by immunofluorescence microscopy. The colocalization intensity was analyzed using Pearson’s correlation coefficient using ImageJ software. Scale bar: 20 µm. (**D**) L929 cells were retrovirally transfected with cDNA encoding mCitrine-LC3B. Cells were left untreated (medium, M) or treated using 10 ng/ml TNF + 30 µM QVD (TQ) or EBSS with or without 20 nM bafilomycin A_1_ (B) for indicated times. Cells were collected and mCitrine fluorescence intensity was measured by flow cytometry. The mean of fluorescence intensity for each sample was normalized to cells incubated in growth medium (M). Data represent mean + SD from two independent experiments. (**A-D**) Statistical analysis was done by repeated measures two-way ANOVA (corrected by Tukey’s multiple comparisons test) for A, B and D, and by ordinary one-way ANOVA (corrected by Tukey’s multiple comparisons test) for C. For C, statistical analysis was additionally performed using unpaired t test with Welch’s correction (TQ treatment of non-targeting vs. *Ripk3* siRNA). For A and B, statistically significant differences are only indicated for 6 h; for D, statistically significant differences are only indicated for 6 h vs. 6 h + bafilomycin A_1_. Statistically significant differences to control (medium, M) are depicted as letters directly above the bars. * or *a: P* < 0.05, ** or *b: P* < 0.01, *** or *c: P* < 0.001, **** or *d: P* < 0.0001

Generally, the above-described formation of ATG14 or ATG16L1 puncta upon TQ treatment might also be caused by a blockade of the autophagic flux. Accordingly, we also aimed at investigating later steps of the autophagic pathway. Following TQ treatment, we monitored levels of MAP1LC3/LC3-II, which is the phosphatidylethanolamine-conjugated form of LC3 and represents a marker protein for autophagosomes. We observed that LC3-II levels increased with time upon TQ treatment (Figure 5A). TQ-induced LC3-II levels did not further increase with simultaneous bafilomycin A_1_ treatment, indicating that lysosomal LC3-II (and thus autophagosome) degradation was rather blocked than induced upon TNF-induced necroptosis (Figure 5A).

Next to LC3 turnover, we also analyzed levels of the autophagy receptors SQSTM1/p62 and TAX1BP1, respectively. Of note, these autophagy receptors decreased upon TQ treatment, and this reduction was sensitive to bafilomycin A_1_ treatment (Figure 5A). It has previously been observed that starvation induces the rapid degradation of autophagy receptors by endosomal microautophagy, i.e., a lysosome-dependent but macroautophagy-independent pathway [52]. To further evaluate this observation, we made use of the PIK3C3/VPS34 inhibitor SAR405, which blocks early steps of macroautophagy. Indeed, autophagy receptors still became degraded in the presence of SAR405 (Figure S3A), indicating that this reduction is not caused by the execution of macroautophagy. Previously, the starvation-induced degradation of autophagy receptors by endosomal microautophagy has been associated with late endosomes including multivesicular bodies (MVBs) [52]. Interestingly, we also observed MVB-like structures in L929 cells treated with TQ (Figure S3B). Furthermore, the degradation of autophagy receptors by endosomal microautophagy was reported to depend on the endosomal sorting complex required for transport III (ESCRT-III) component CHMP4B (charged multivesicular body protein 4B) [52]. Similarly, we observed that the TQ-induced degradation of SQSTM1/p62 and TAX1BP1 was abolished upon siRNA-mediated depletion of CHMP4B (Figure S3C).

The TQ-induced accumulation of LC3-II was clearly dependent on RIPK3-dependent necroptosis, since we did not detect LC3-II accumulation in RIPK3- or MLKL-deficient L929 cells (Figure 5B), or in cells treated with *Ripk3* siRNA or the RIPK3 inhibitor GSK’872 (Figures S4A and S4B). In order to confirm our observation of blocked LC3 turnover by an independent approach, we performed LC3 immunofluorescence in L929 cells. Treatment with TQ increased LC3-positive puncta (Figure S4C). Similar to the LC3 turnover analysis by immunoblot, this increase in LC3 puncta was not further increased by parallel bafilomycin A_1_ treatment but was reduced by RIPK3 knockdown. To further confirm our observation that the autophagic flux is indeed blocked on the lysosomal level, we made use of the mRFP-EGFP-rLC3 tandem construct [53,54]. The GFP signal is sensitive to the acidic and/or proteolytic environment of lysosomes, whereas the mRFP signal is more stable [53,54]. We detected a strong accumulation of the tandem fluorescence-tagged LC3 upon TQ or medium/bafilomycin A_1_ treatment (Figure 5C), and again the TQ-induced mRFP-EGFP-rLC3 accumulation was reduced upon siRNA-mediated knockdown of RIPK3 (Figure 5C). Finally, we assessed autophagic flux by monitoring mCitrine-LC3 degradation by flow cytometry. Whereas starvation induced a clear reduction of mCitrine-LC3 levels, mCitrine-dependent fluorescence remained unaltered or even increased upon TQ treatment (Figure 5D). Taken together, we showed by different assays that TQ-induced necroptosis blocks lysosomal autophagosome degradation.

### TNF inhibits autophagic flux via dysregulating SNARE-mediated autophagosome-lysosome fusion

**Figure 6.**
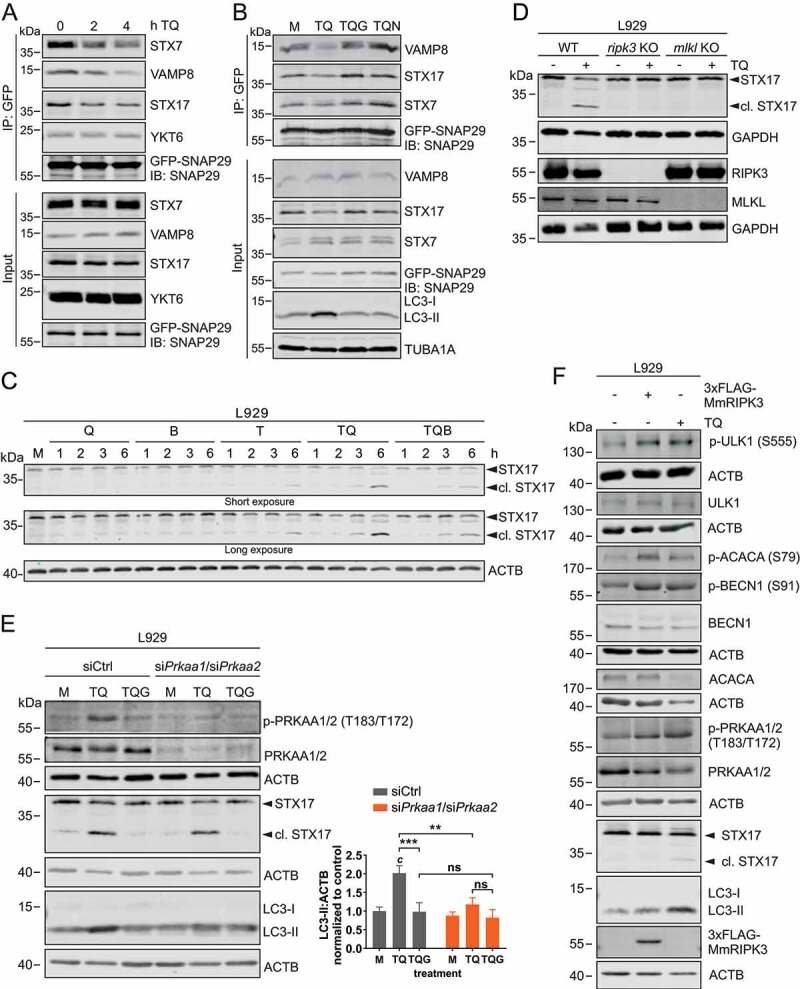
Necroptosis induced by TNF destabilizes SNARE complexes and cleaves STX17 to block LC3 degradation. (**A**) L929 cells stably expressing GFP-SNAP29 were exposed to 10 ng/ml TNF and 30 µM QVD (TQ) for indicated times. Then, cells were lysed and cleared cellular lysates were subjected to immunopurification using anti-GFP beads. Purified proteins were subjected to SDS-PAGE and analyzed by immunoblotting for indicated proteins. (**B**) L929 cells stably expressing GFP-SNAP29 were left untreated (medium, M) or exposed to 10 ng/ml TNF + 30 µM QVD (TQ), TQ plus 5 µM GSK’872 (TQG), or TQ plus 5 µM necrostatin-1 (TQN) for 4 h. Then, cells were lysed and cleared cellular lysates were subjected to immunopurification using anti-GFP beads. Purified proteins were subjected to SDS-PAGE and analyzed by immunoblotting for indicated proteins. (**C**) L929 cells were left untreated (medium, M) or exposed to 30 µM QVD (Q), 20 nM bafilomycin A_1_ (B), 10 ng/ml TNF (T), 10 ng/ml TNF + 30 µM QVD with or without 20 nM bafilomycin A1 (TQ or TQB) for indicated times. Then, cells were lysed and cleared cellular lysates were subjected to SDS-PAGE and immunoblotting for STX17 and ACTB. (**D**) L929 WT, *ripk*3 KO or *mlkl* KO cells were left untreated or exposed to 10 ng/ml TNF + 30 µM QVD (TQ) for 4 h. Then, cells were lysed and cleared cellular lysates were subjected to SDS-PAGE and immunoblotting for STX17, RIPK3, MLKL, and GAPDH. (**E**) L929 cells were transfected with non-targeting (siCtrl) or *Prkaa1/Prkaa2* siRNAs (si*Prkaa1/*si*Prkaa2*). 48 h post transfection, cells were left untreated (medium, M) or exposed to 10 ng/ml TNF + 30 µM QVD (TQ) with or without 5 µM GSK’872 (G) for 4 h. Then, cells were lysed and cleared cellular lysates were subjected to SDS-PAGE and analyzed by immunoblotting for indicated proteins. The density of each protein band was divided by the average of the density of all bands from the same protein on the membrane. Fold changes were calculated by dividing each normalized ratio (protein to loading control) by the average of the ratios of the control lane (siCtrl, medium). Statistical graphics represents mean + SD (n = 3). Statistical analysis was done by ordinary two-way ANOVA (corrected by Tukey’s multiple comparisons test). Statistically significant differences are only indicated for TQ and TQG (scr vs. *Prkaa1/Prkaa2* siRNA) and for TQ vs. TQG (within each treatment). Statistically significant differences to control (siCtrl, medium) are depicted as letters directly above the bars. ** or *b: P* < 0.01, *** or *c: P* < 0.001, ns: non-significant. (**F**) L929 cells were left untransfected or transiently transfected with cDNA encoding 3xFLAG-MmRIPK3 for 24 h. Then untransfected cells were left untreated or exposed to 10 ng/ml TNF + 30 µM QVD (TQ) for 3 h. Cells were lysed and cleared cellular lysates were subjected to SDS-PAGE and immunoblotting for indicated proteins

In order to characterize the lysosomal blockade described above, we first measured CTSB (cathepsin B) and CTSL (cathepsin L) activities. However, we did not detect any differences between TQ-treated or control cells, whereas this was clearly the case for bafilomycin A_1_ treatment (Figure S5). The fusion of autophagosomes with lysosomes is a critical step during the autophagic pathway, and this step is mediated—among other factors—by autophagosomal and lysosomal SNARE proteins. First, we investigated the stability of STX17-SNAP29-VAMP8 and YKT6-SNAP29-STX7 complexes, respectively, since these SNARE complexes have been implicated in the autophagosome-lysosome fusion process [55,56]. For that, we overexpressed GFP-SNAP29 in L929 cells and investigated the interactions with the SNARE proteins upon TQ treatment (Figure 6A). We observed reduced binding to VAMP8, STX17 and STX7 upon treatment with TQ. These data indicate that the SNARE complexes responsible for the fusion of autophagosomes with lysosomes become destabilized upon TQ treatment. Furthermore, we also observed that the destabilization of these interactions could be prevented by RIPK1 and RIPK3 inhibitors, respectively (Figure 6B). We also analyzed general STX17 expression upon TQ treatment. We observed that this treatment induces the reduction of full-length STX17 (Figure 6C). Furthermore, we simultaneously observed the appearance of an additional band at a lower molecular weight, suggesting a possible cleavage of STX17 (Figure 6C). This TQ-induced STX17 fragment was also observed for overexpressed GFP-STX17 (Figure S6A). Furthermore, we confirmed that the detected fragment derives from STX17 by siRNA (Figure S6B). The appearance of this truncated STX17 was clearly dependent on the execution of necroptosis, since we did not observe this fragment in *ripk3* or *mlkl* KO L929 cells (Figure 6D) or in cells treated with GSK’872 (Figure 6E). In contrast, the STX17 fragment was detectable in cells with a knockdown of PRKAA1/2 (Figure 6E), indicating that induction of early autophagy events via the RIPK3-AMPK and the presumable cleavage of STX17 are independent events. This was also confirmed by two alternative approaches. First, we overexpressed 3xFLAG-MmRIPK3 in wild-type L929 cells. In these cells, phosphorylation of AMPK and of its downstream substrates was induced, whereas the shorter STX17 fragment was not detected (Figure 6F). Second, the combination of AMPK activating compounds with bafilomycin A_1_ induced AMPK activation, phosphorylation of AMPK substrates, and LC3 accumulation (Figure S6C). However, neither STX17 was cleaved nor cell death induced upon this treatment (Figure S6C and S6D). Taken together, early autophagy events can be mediated by RIPK3, but additional pro-necroptotic, TNF-dependent signaling events are required for the dysregulation of SNARE-mediated autophagosome-lysosome fusion.

## Discussion

Autophagy, apoptosis and necroptosis are three main stress responses regulating life and death of a cell. Whereas the crosstalk between autophagy and apoptosis is well established, the relationship between autophagy and necroptosis awaits further clarification. Although recent reports suggest the existence of this crosstalk [57–60], mechanistic details are rudimentary. Here, we report that necroptosis induced by TNF blocks late events of the autophagic pathway (i.e. the degradation of autophagosomes by lysosomes) at least in part via the dysregulation of SNARE-mediated autophagosome-lysosome fusion. We also found that early signaling events of autophagy—for example AMPK-dependent phosphorylation of ULK1 and BECN1 or formation of ATG14 or ATG16L1 puncta—are induced upon TQ or TSZ treatment. Remarkably, we found that the pro-necroptotic RIPK3 directly phosphorylates PRKAA1/2 at T183/T172 and thus activates it. This is especially noteworthy since only limited number of examples exist for both RIPK3 substrates and AMPK-activating kinases.

At first glance, the promotion of early autophagy events and the simultaneous inhibition of late events appear to be contradictory. We observed that autophagy—specifically the lysosomal degradation of autophagosomes—is blocked upon TQ treatment and necroptosis induction. Additionally, we observed that the lysosomal CTSB and CTSL remain functional upon TQ treatment, which was not the case for bafilomycin A_1_ treatment. We found that the blockade of autophagosome degradation is—likely among additional mechanisms—caused by the dysregulation of proteins that participate in the fusion of autophagosomes with lysosomes. Specifically, we observed destabilized interactions between VAMP8, STX17, STX7 and SNAP29, a reduction of full-length STX17, and the appearance of a shorter STX17-derived fragment upon TQ treatment. An increased lipidation of LC3 due to the inhibition of the autophagic flux has also been postulated by Frank et al. recently [57]. They postulate that activated MLKL has to associate with intracellular membranes in order to inhibit autophagy [57]. Further studies will have to determine whether this function of MLKL is connected to the observed STX17 modification. However, we found that the presence of RIPK3 and MLKL are clearly required for both LC3 accumulation and STX17 fragmentation upon TQ treatment. Generally, proteolytic events control several forms of regulated necrosis [61]. Since our stimuli contain the caspase inhibitors QVD or zVAD, we can exclude the involvement of caspases in the proteolytic cleavage of STX17. It is well established that inhibitors of serine proteases such as tosyl phenylalanyl chloromethyl ketone (TPCK) can block necroptosis [61], but the specific substrates that become cleaved during necroptosis await further clarification. In our case, we could not block STX17 truncation upon TQ treatment using TPCK (data not shown). Nevertheless, the dysregulation of SNARE-mediated autophagosome-lysosome fusion and the resulting blockade of autophagy has been observed for other stimuli, e.g. enteroviral infections [62,63].

Interestingly, we observed the degradation of the selective autophagy receptors SQSTM1/p62 and TAX1BP1 upon TQ treatment, although autophagosome degradation is blocked. We think that this turnover is caused by a macroautophagy-independent mechanism, and this is clearly supported by our experiments using the PIK3C3/VPS34-specific inhibitor SAR405. It has previously been described that starvation induces the rapid degradation of selective autophagy receptors by endosomal microautophagy [52]. Although we do not necessarily think that these are identical phenomena, it is noteworthy that also TQ treatment induces the accumulation of MVBs and that the degradation of autophagy receptors was abolished upon knockdown of CHMP4B (Figures S3B and S3C). In contrast to the LC3 levels reported for the immediate response to starvation [52], we observed a TQ-induced accumulation of LC3. Notably, we did not observe LC3-II accumulation in cells treated with TQ plus SAR405 and bafilomycin A_1_, whereas this was clearly the case for the combination of TQ with bafilomycin A_1_ alone (Figure S3A). We speculate that TQ plus SAR405 represents a strong cell death stimulus, which might also induce non-canonical and specifically PIK3C3/VPS34-independent LC3 lipidation [64]. In these pathways, LC3 conjugation occurs at single membranes [65]. For example, it has been reported that drugs possessing lysosomotropic or ionophore drugs are able to induce LC3 lipidation at single-membrane compartments of the endolysosomal pathway [66–68]. It has also been suggested that these non-canonical LC3 lipidations require vacuolar-type H^+^-ATPase (V-ATPase) activity [67,69], and accordingly treatment with bafilomycin A_1_ in combination with TQ and SAR405 causes a decrease of LC3-II [69]. The strong cell death induction by the combination of TQ and SAR405 can also be deduced from the bafilomycin A_1_-insensitive degradation of the selective autophagy receptors SQSTM1/p62 and TAX1BP1, suggesting a caspase- and lysosome-independent demise of these proteins during cell death. Howsoever, in summary we observed a TNF-induced blockade of autophagosome degradation, and the dysregulation of SNARE-mediated autophagosome-lysosome fusion likely contributes to this blockade.

Generally, the blockade of cyto-protective pathways upon necroptosis induction is comprehensible and is similar to the crosstalk between apoptosis and autophagy, in which e.g. activated caspases cleave several autophagy-relevant proteins and thus inactivate their autophagic function [70]. In contrast, the induction of early autophagy signaling events upon TQ or TSZ treatment is less intuitive. We speculate that this phenomenon might be explained by two hypotheses: 1) an anti-necroptotic and thus cyto-protective function of RIPK3, or 2) a pro-necroptotic function of early autophagy events. In the first scenario, it might be that RIPK3 regulates cyto-protective autophagy independently of its pro-death function during necroptosis. So far, RIPK3 has been attributed a central role in necroptosis signaling, and the best-characterized RIPK3 substrate MLKL is a main executor of necroptosis [31,32,39]. A possible role of RIPK3 in the regulation of autophagy has also been suggested by two other groups. Harris et al. suggest RIPK3 positively regulates autophagy since depletion of RIPK3 inhibits autophagic flux and leads to the accumulation of autophagosomes and amphisomes [59]. In contrast, Matsuzawa et al. reported that RIPK3 serves as a negative regulator of selective autophagy by binding to SQSTM1/p62 and thus regulating SQSTM1/p62-LC3 complex formation [71]. We observed that RIPK3 positively regulates AMPK activity. Activated AMPK phosphorylates both ULK1 and BECN1, and these two phosphorylation events have been associated with a positive regulation of autophagy [10,16,41,42]. We also observed increased ATG14 and ATG16L1 puncta formation in a RIPK3-dependent manner, although we cannot exclude that these puncta formations are caused by the TNF-mediated blockade of autophagosome degradation. The RIPK3-dependent induction of early autophagy signaling events might pursue a cyto-protective function and slow down the execution of necroptosis. Along these lines, necroptosis-inhibitory functions have been attributed to AMPK and to ULK1 [43–45], and this is confirmed by our data (Figure S2B-D).

In a second and opposing scenario, one might speculate that the induction of early autophagy signaling events supports necroptosis. Goodall et al. reported that inhibition of late stage autophagy enhanced TNFSF10/TRAIL-induced cell death. In contrast, genetic or pharmacological inhibition of early/mid-stage autophagy prevented cell death [58,72]. The authors hypothesized that components of the autophagy machinery mediate cell death by functioning as a scaffold for necrosome formation and activation. Specifically, they propose that the interaction between SQSTM1/p62 and RIPK1 localizes the necrosome on the autophagosome [58,72]. It is tempting to speculate that RIPK3 itself promotes early autophagy events to support its pro-necroptotic function. Furthermore, Goodall et al. also stated that apparently the turnover of cellular components does not mediate this pro-death function and that they observed necrosome structures on quite mature autophagosomes [58,72]. Our observed blockade of autophagosome degradation might point toward the same direction. We observed increased ATG14-, ATG16L1- or LC3-positive structures, which potentially reflect increased necrosome-activating platforms. Collectively, our observations might indicate that RIPK3 “feeds” the autophagic flux via AMPK-dependent phosphorylation of ULK1 and BECN1 in order to ensure sufficient autophagosome maturation and accumulation. AMPK becomes activated under stress and starvation conditions, but it has also been shown that AMPK can support autophagy under nutrient sufficiency [29]. Accordingly, it is conceivable that AMPK also supports autophagy induction upon TQ/TSZ treatment. Admittedly, our second model is rather speculative and especially kinetic and spatial aspects need further validation. As mentioned above, necroptosis is rather increased in *ulk1 ulk2* or *prkaa1 prkaa2* DKO MEFs compared to wild-type control cells, supporting a generally anti-necroptotic function of these two kinases.

AMPK is the central energy sensor of the cells. To date, three kinases have been shown to phosphorylate and thus activate AMPK, i.e. STK11, CAMKK2, and MAP3K7 [2–4,24–27]. Here, we showed that RIPK3 is another AMPK-activating kinase. However, we do not believe that RIPK3-dependent phosphorylation is the only mechanism to activate AMPK during necroptosis. We have assessed the relevance of the AMP-binding site 3 in PRKAG1, since it has previously been described that the induction of necroptosis results in reduced cellular ATP levels [49,50]. We did not observe any difference in PRKAA1/2 phosphorylation between cells expressing either wild-type PRKAG1 or the R299G variant. However, additional experiments are required to fully elucidate the mechanisms leading to AMPK activation during necroptosis. Nevertheless, we think that the RIPK3-dependent activation of AMPK represents another level of crosstalk between necroptosis and autophagy. The ultimate fate of a cell under stress conditions is determined by the integration of different cellular stress responses. Accordingly, a deeper understanding of the interplay between these stress responses is necessary in order to exploit these pathways for potential therapeutic approaches.

## Materials and Methods

### Antibodies and reagents

Anti-mouse RIPK3 (ProSci, 2283), anti-MLKL phospho-Ser345 [EPR9515(2)] (Abcam, ab196436), anti-MLKL (Sigma-Aldrich, SAB1302339), anti-FLAG (Sigma-Aldrich, F3165), anti-ACTB/β-actin (Sigma-Aldrich, A5316), anti-PRKAA1/2 (Cell Signaling Technology, 2793 (clone F6) and 2603 (clone 23A3) for immunoblot, and Santa Cruz Biotechnology, sc-74,461 (clone D-6) for immunopurification), anti-PRKAA1/2 phospho-T183/T172 (40H9) (Cell Signaling Technology, 2535), anti-GST (Sigma-Aldrich, G7781), anti-LC3B (Cell Signaling Technology, 2775 for immunoblot [detects endogenous levels of total LC3B protein; cross-reactivity may exist with other LC3 isoforms according to manufacturer specification], and MBL International; PM036 for immunofluorescence [reacts with LC3A/LC3B/LC3C according to manufacturer specification]), anti-BECN1 (H-300) (Santa Cruz Biotechnology, sc-11,427), anti-BECN1 phospho-S93 (D9A5G) (Cell Signaling Technology, 14717S), anti-ATG14 (Santa Cruz Biotechnology, sc-164767), anti-ACACA phospho-S79 (Cell Signaling Technology, 3661), anti-ACACA (Cell Signaling Technology, 3662), anti-ULK1 phospho-S555 (Cell Signaling Technology, 5869), anti-ULK1 (Cell Signaling Technology, 8054), anti-ATG16L1 (MBL International, PM040), anti-STX17 (Sigma-Aldrich, HPA001204), anti-TAX1BP1 (Proteintech, 14424-1-AP), anti-anti-SQSTM1/p62 (C terminus) (PROGEN, GP62-C), anti-TUBA1A/tubulin (Sigma-Aldrich, T5168), anti-GAPDH (Abcam, ab8245), anti-CHMP4B (Cell Signaling Technology, 42466), anti-SNAP29 [EPR9198(2)] (Abcam, ab181151), anti-VAMP8 (Cell Signaling Technology, 13060), anti-STX7 (Bethyl Laboratories, A304-512A-M), anti-YKT6 (Santa Cruz Biotechnology, sc-365732), anti-GFP (Nacalai USA, 04404–84), anti-ATG13 (MBL International, M183-3). IRDye 800- or IRDye 680-conjugated secondary antibodies were purchased from LI-COR Biosciences (925–32212/13/14, 925–68072/73/74). Alexa Fluor® 647-conjugated goat anti-mouse IgG (H + L) (115–605-003), Alexa Fluor® 647-conjugated goat anti-rabbit IgG (H + L) (111–605-003) and Alexa Fluor® 488-conjugated goat anti-rabbit IgG (H + L) (111–545-003) antibodies were purchased from Jackson ImmunoResearch Laboratories. Bafilomycin A_1_ (Alfa Aesar, J61835), TNF Recombinant Mouse Protein (Thermo Scientific, PMC3014), active GST-HsRIPK3 (Sigma-Aldrich, SRP5316), necrostatin-1 (Enzo Life Sciences, BML-AP309-0020), GSK’872 (Calbiochem, 530389), MK8722 (MedChemExpress, HY-111363), PF739 (AOBIOUS, AOB33584), Alkaline Phosphatase (Thermo Fisher, EF0651), Q-VD-OPh (Selleck Chemicals, S7311), z-VAD-FMK (Selleck Chemicals, S7023), ProLong® Gold Antifade Reagent with DAPI (Cell Signaling Technology, 8961), Lipofectamine^TM^ RNAiMAX Transfection Reagent (Thermo Fisher, 13778150; for siRNA transfection), FuGENE® 6 (Promega, E2692; for transfection of Plat-E and HEK293), Lipofectamine™2000 (Thermo Fisher, 11668027; for transfection of L929), Magic Red Cathepsin-B/L Assay (ImmunoChemistry Technologies, 937/941), Dorsomorphin (PRKAA1/2 inhibitor; Sigma-Aldrich, P5499), SAR405 (Selleck Chemicals, S7682). His-AMPK [His-HsPRKAA1 (11–559) + HsPRKAB2 (1–272) + HsPRKAG1 (1–331)] (DU32489) was obtained through the MRC PPU Reagents and Services facility (MRC PPU, College of Life Sciences, University of Dundee, Scotland, mrcppureagents.dundee.ac.uk).

### Constructs and siRNAs

pBOB-FLAG-MmRIPK3 plasmid was kindly provided by Jiahuai Han (School of Life Sciences, Xiamen University, China). pCI-neo-3xFLAG-HsRIPK3 was kindly provided by Sudan He (Cyrus Tang Hematology Center, Soochow University, China). pMRXIP-GFP-STX17 and pMRXIP-GFP-SNAP29 were kindly provided by Noboru Mizushima (Department of Biochemistry and Molecular Biology, Graduate School and Faculty of Medicine, The University of Tokyo, Tokyo, Japan; Addgene, 45909 and 45923; http://n2t.net/addgene:45909 and http://n2t.net/addgene:45923; RRID:Addgene_45909 and RRID:Addgene_45923) and have been previously described [55]. pMSCVblast-mCitrine-HsLC3 and pMSCVblast-mRFP-EGFP-rLC3 were previously described [46]. pCI-neo-3xFLAG-HsRIPK3^K50A^ (kinase dead) plasmid was generated using one step PCR from pCI-neo-3xFLAG-HsRIPK3. cDNA encoding MmRIPK3 harboring *XhoI* and *NotI* restriction enzyme sites was amplified from pBOB-FLAG-MmRIPK3 plasmid. PCR fragment was inserted into pGEX-5X-3 (GE Healthcare Life Sciences, 28–9545-55) and generated pGEX-5X-3-MmRIPK3. cDNA encoding MmRIPK3 harboring *XhoI* and *NotI* restriction enzyme sites amplified from pBOB-FLAG-MmRIPK3 plasmid was inserted into pCI-neo-3xFLAG-HsRIPK3 vector digested with *XhoI* and *NotI* restriction enzymes to generate pCI-neo-3xFLAG-MmRIPK3. cDNA of full length 3xFLAG-MmRIPK3 harboring *BglII* and *NotI* restriction enzyme sites amplified from pCI-neo-3xFLAG-MmRIPK3 was inserted into pMSCVpuro vector to generate pMSCVpuro-3xFLAG-MmRIPK3. cDNA encoding HsPRKAA1 was cloned from HEK293 cDNA and inserted into pGEX-5X-3. cDNAs of truncated HsPRKAA1 (1–278) and HsPRKAA1 (279–559) harboring *XhoI* and *NotI* restriction enzyme sites were amplified from pGEX-5X-3-HsPRKAA1 and inserted into pGEX-5X-3. cDNA of GST-HsPRKAA1 (1–278)^T183A^ was amplified from GST-HsPRKAA1 (1–278) using one-step PCR. cDNA encoding HsPRKAG1 was cloned from HEK293 cDNA and inserted into pGEX-3X (GE Healthcare Life Sciences, 28–9546-54) to generate pGEX-3X-HsPRKAG1 harboring *BamHI* and *EcoRI* restriction enzyme sites. pGEX-3X-HsPRKAG1^R299G^ mutant was generated using site-directed mutagenesis based on pGEX-3X-HsPRKAG1. Then, cDNA encoding HsPRKAG1 WT or HsPRKAG1^R299G^ mutant was amplified from the corresponding templates pGEX-3X-HsPRKAG1 WT or R299G and inserted into pCI-neo-3xFLAG derived from pCI-neo-3xFLAG-MmRIPK3 digested with *XhoI* and *NotI* restriction enzymes using sequence and ligation-independent cloning and generated pCI-neo-3xFLAG-HsPRKAG1 WT or HsPRKAG1^R299G^ mutant. Mouse *Ripk3* (ON-TARGETplus siRNA SMARTpool, L-049919-00-0010), mouse *Stx17* (ON-TARGETplus siRNA SMARTpool, L-049195-01-0005) and control siRNAs (ON-TARGETplus non-targeting pool, D-001810-10-20) were obtained from Dharmacon (working concentration: 20 nM). Mouse *Prkaa1* siRNA (s98534), mouse *Prkaa2* siRNA (s99116) and mouse *Chmp4b* (s93709) were obtained from Thermo Fisher (working concentration: 20 nM).

### Cell lines

*ripk3* KO MEFs, *ripk3* KO L929 and *mlkl* KO L929 cells have previously been described and were kindly provided by Jiahuai Han (School of Life Sciences, Xiamen University, China) [73,74]. *ulk1 ulk2* DKO MEFs have previously been described and were kindly provided by Tullia Lindsten (Memorial Sloan Kettering Cancer Center, New York, USA) [75]. The *prkaa1 prkaa2* DKO MEFs have previously been described and were kindly provided by Benoit Viollet (Inserm, U1016, Institut Cochin, 75,014 Paris, France) [76]. For the generation of L929 cells (kindly provided by Sebastian Wesselborg, Institute of Molecular Medicine I, Heinrich Heine University, Düsseldorf, Germany) stably expressing mRFP-EGFP-rLC3, mCitrine-HsLC3 or GFP-STX17 and *ripk3* KO MEFs stably expressing 3xFLAG-MmRIPK3, the vectors pMSCVblast-mRFP-EGFP-rLC3, pMSCVblast-mCitrine-HsLC3, pMRX-IP-GFP-STX17 or pMSCVpuro-3xFLAG-MmRIPK3 were transfected into Plat-E cells (kindly provided by Toshio Kitamura, Institute of Medical Science, University of Tokyo, Japan) using FuGENE® 6 (Promega, E2692). After 48 h, retroviral supernatant was collected and used for the infection of L929 cells or MEFs in combination with 10 µg/ml polybrene (Sigma-Aldrich, H9268-106). After 12 h, cells were selected in medium containing puromycin (50 µg/ml for L929 and 2.5 µg/ml for MEFs) or 35 µg/ml blasticidine. MEFs, L929 and Plat-E cell lines were cultured in high D-glucose DMEM (Thermo Fisher, 41965–039) supplemented with 10% FCS (Thermo Fisher, 10270–106), 100 U/ml penicillin and 100 μg/ml streptomycin at 37°C and 5% CO_2_. For amino acid starvation, cells were washed once with Dulbecco’s Phosphate-Buffered Saline (DPBS; Thermo Fisher, 14190094) and incubated for the indicated time points in Earle’s Balanced Salt Solution (EBSS; Thermo Fisher, 24010043).

### Immunofluorescence and proximity ligation assay (PLA)

Cells were seeded on glass coverslips overnight and exposed to indicated treatments at the next day. Then, cells were fixed using 4% paraformaldehyde for 10 min at room temperature and after washing three times with DPBS, cells were permeabilized with 50 µg/ml digitonin (Sigma-Aldrich, D141) for 5 min at room temperature. Cells were again washed with DPBS three times and incubated with 3% BSA (Roth, 8076) for 30 min at room temperature. The coverslips were transferred to a humidified chamber and incubated with indicated primary antibodies in 3% BSA for 1 h at room temperature. The cells were washed 3 times (3 min each time) with DPBS and incubated with indicated secondary antibodies in 3% BSA for 1 h at room temperature in the dark. The cells were washed 3 times (3 min each time) with DPBS and mounted on slide glass with ProLong® Gold Antifade Reagent with DAPI after rinsing the coverslips briefly in distilled water. After drying the coverslips, they were stored at 4°C. For proximity ligation assay, fluorescence signals were measured according to Duolink® PLA Fluorescence Protocol (Sigma-Aldrich, DUO82005 [anti-rabbit] and DUO82001 [anti-mouse]). Images were captured by Zeiss Axio Observer 7 (ApoTome extension, Objective Plan-Apochromat 40x/1,4 Oil DIC M27). For all immunofluorescence and PLA analyses, puncta quantification was done using ImageJ software.

### Protein expression and purification

pGEX-5X-3-HsPRKAA1 variants and pGEX-5X-3-MmRIPK3 were transformed into *E. coli* BL21 (DE3). Target protein expression was induced by adding 0.1–1 mM IPTG (Sigma-Aldrich, I5502) for 3–5 h at 30°C. Bacteria were harvested and resuspended in bacterial lysis buffer (300 mM NaCl, 50 mM Tris-HCl, pH 8.0, 5 mM EDTA, 0.01% Nonidet P-40/IGEPAL CA-630 [Sigma-Aldrich, I3021], 100 ng/ml lysozyme [Sigma-Aldrich, L166], 1x protease inhibitor cocktail [Sigma-Aldrich, P2714]). After sonification and centrifugation at 10,000 x g for 20 min, the supernatant was incubated with glutathione sepharose 4B beads (GE Healthcare Life Sciences/Cytiva, 17–0756-01) overnight. After washing 3 times with lysis buffer, protein bound to beads were stored at −80°C or soluble proteins were obtained by incubation of the beads with elution buffer (50 mM Tris-HCl, pH 8.0, 20 mM glutathione reduced [Roth, 6832.4]) for 10 min at room temperature (3–5 times). Purified proteins were stored at −80°C.

### In vitro kinase assay

For *in vitro* kinase assays, 1–2 μg substrate were incubated with 0.25–0.5 μg activated kinase in kinase buffer (50 mM Tris-Cl, pH 7.5, 0.1 mM EGTA, 1 mM DTT, 7.5 mM Mg[CH_3_COO]_2_, 2 µM ATP [non-radioactive; Cell Signaling Technology, 9804] with or without 10 μCi [γ-^32^P]-ATP [Hartmann Analytics, SRP 301]) for 45 min at 30°C. The kinase reaction was stopped by adding loading buffer and boiling for 5 min at 95°C. After Coomassie Brilliant Blue staining or immunoblotting, autoradiography was performed or phosphorylation was detected using anti-PRKAA1/2 phospho-T183/T172 antibody.

### Protein dephosphorylation assay

Two duplicate samples were separated by the same SDS-PAGE and then transferred to the same PVDF membrane. Following blocking with 5% BSA in TBS-T (10 mM Tris-HCl, pH 7.6, 150 mM NaCl, 0.05% Tween-20 [Sigma-Aldrich, P1379]) for 1 h at room temperature, the PVDF membrane was washed with TBS-T twice (5 min each). The membrane was cut into two halves with duplicate samples on each membrane. The two membranes were placed into two tubes with or without 10 U alkaline phosphatase (2 ml reaction volume) and incubated for 1 h at 37°C with shaking (350 rpm). After washing twice with TBS-T (5 min each), the membranes were incubated with the corresponding primary antibodies at 4°C overnight.

### Immunoblotting, immunopurification and size exclusion chromatography

For immunoblotting, cells were lysed in 1% Nonidet P-40/IGEPAL CA-630 lysis buffer (50 mM Tris-Cl, pH 7.5, 150 mM NaCl, 1 mM EDTA, 1% Nonidet P-40/IGEPAL CA-630, 10% glycerol, 1 mM Na_3_VO_4_, 50 mM NaF and protease inhibitor cocktail [Sigma-Aldrich, P2714]). Alternatively, whole cell lysates were prepared by direct addition of sample buffer (125 mM Tris-Cl, pH 6.8, 17.2% glycerol, 4.1% SDS, 0.02% bromophenol blue and 2% β-mercaptoethanol) to the cells and subsequent sonification. An equal amount of protein (30–50 μg) were separated by SDS-PAGE, and then transferred to PVDF membrane (Millipore, IPFL00010). Target proteins were detected by the indicated primary antibodies, followed by the corresponding IRDye®800- or IRDye®680-conjugated secondary antibodies (LI-COR Biosciences). Signals were detected with an Odyssey Infrared Imaging system (LI-COR Biosciences). For immunopurification, cells were lysed in the same lysis buffer for 30 min on ice. After 10,000 x g centrifugation for 10 min, the supernatants were incubated with indicated beads [FLAG® M2 Affinity Gel (Sigma-Aldrich, A2220) or Protein A/G PLUS-Agarose (Santa Cruz Biotechnology, sc-2003) conjugated with anti-PRKAA1/2 antibodies (Santa Cruz Biotechnology, sc-74461)] overnight. Thereafter, the beads were washed five times with lysis buffer, and boiled in sample buffer for 5 min at 95°C. Purified proteins were analyzed by immunoblotting. Size exclusion chromatography was performed as described previously [48].

### GST affinity purification

GST fusion proteins were purified from *E. coli* BL21 (DE3) with glutathione sepharose 4B beads (GE Healthcare Life Sciences/Cytiva, 17–0756-01). For GST affinity purfication, 4 µg of GST fusion protein were incubated with 1 µg His-AMPK [His-HsPRKAA1 (11–559) + HsPRKAB2 (1–272) + HsPRKAG1 (1–331)] in 0.5% Nonidet P-40/IGEPAL CA-630 lysis buffer (50 mM Tris, pH 8.0, 100 mM NaCl, 6 mM EDTA, 6 mM EGTA, 0.5% Nonidet P-40/IGEPAL CA-630, 1 mM dithiothreitol supplemented with protease inhibitor cocktail) at 4°C overnight and then washed five times with lysis buffer. The beads were boiled with sample buffer at 95°C for 5 min and purified proteins were analyzed by immunoblotting.

### Cell death assay

Total cells treated with indicated stimuli were trypsinized and collected. Cells were then incubated in propidium iodide (PI) solution (5 µg/ml in DPBS) at 4°C for 1 h. PI-positive cells were measured by flow cytometry (LSRFortessa^TM^, BD Biosciences).

### Transmission electron microscopy (TEM)

L929 cells were seeded into 10 cm dishes and incubated in DMEM medium including 10% FCS overnight. After indicated treatments, cells were washed once using 1x DPBS. Then, cells were fixed using 3% glutaraldehyde in 0.1 M sodium cacodylate buffer, pH 7.2 for 2 h at room temperature (RT) in the hood. Then, cells were harvested with a cell scraper and centrifuged at approx. 4000 x g for 5 min. Pellets were washed twice with 0.1 M sodium cacodylate buffer, pH 7.2 (centrifugation at 4000 x g for 5 min). The pellets were heated to 40°C and embedded into 3% low melting agarose. Agarose was dissolved at 40°C in a water bath. The supernatant was aspirated and a volume of approx. 10 µl was left. The pellet was resuspended using the same volume of agarose. The mixture (agarose + pellet) was centrifuged at approx. 15,000–20,000 x g, 2 min. The samples were covered with 1% OsO_4_ in sodium cacodylate buffer for 50 min, at RT. Then the samples were washed two times with sodium cacodylate buffer for 10 min at RT and once with 70% EtOH for 15 min at RT. Block contrast was applied using 1% uranyl acetate/1% phosphorotungstic acid in 70% EtOH (freshly made and filtered) for 1 h, at RT. The samples were dehydrated using graded ethanol series (90% EtOH, 96% EtOH, 100% EtOH) and embedded in SPURR epoxy resin (Serva, 21050). Polymerization was done at 70°C for 24 h. The 70-nm ultrathin sections were cut using an Ultracut EM UC7 (Leica, Wetzlar, Germany). TEM images were captured using an H7100 TEM (Hitachi, Tokyo, Japan) at 100 V equipped with Morada camera (EMSIS GmbH, Münster, Germany).

### Statistical analysis

For PI uptake, shown data represent at least three independent experiments + SD. Absolute values are shown. For the quantification of immunoblots, the density of each protein band was divided by the average of the density of all bands from the same protein on the membrane. Subsequently, fold changes were calculated by dividing each normalized ratio (protein to loading control) by the average of the ratios of the control lane (as indicated in the corresponding figure legend). For Figure 1A, Figure 5A, Figure 5B, Figure 5D, S1A, S4A and S4B statistical analysis was performed using repeated measures two-way ANOVA (corrected by Tukey’s multiple comparisons test). For Figure 1A, S1A and S4B, statistical analyses were additionally performed using repeated measures two-way ANOVA (corrected by Sidak’s multiple comparisons test). For Figure 2C, Figure 4F, Figure 6C, S2B, S2C, S2D and S3C, statistical analyses were performed using ordinary two-way ANOVA (corrected by Tukey’s multiple comparisons test). For Figure 2A, Figure 2B, Figure 2D, Figure 5C, S4C and S6D, statistical analysis was performed using ordinary one-way ANOVA (corrected by Tukey’s multiple comparisons test). Additionally, for Figure 2B, Figure 3F, Figure 5C and S4C, statistical analysis was performed using an unpaired t test with Welch’s correction. Compared treatments or cell lines are indicated in the corresponding bar diagrams and/or figure legends. *P* values < 0.05 were considered statistically significant. All statistical data were calculated with GraphPad Prism (version 7.01).

## Supplementary Material

Supplemental Material
