# Supplementary material for: TNF-induced necroptosis initiates early autophagy events via RIPK3-dependent AMPK activation, but inhibits late autophagy
Source: Autophagy. 2021 Mar 28;17(12):3992–4009. doi: 10.1080/15548627.2021.1899667 (PMC8726653; doi:10.1080/15548627.2021.1899667)
Supplement: Supplemental Material [file KAUP_A_1899667_SM3553.docx]

**Supplementary Figures**

**
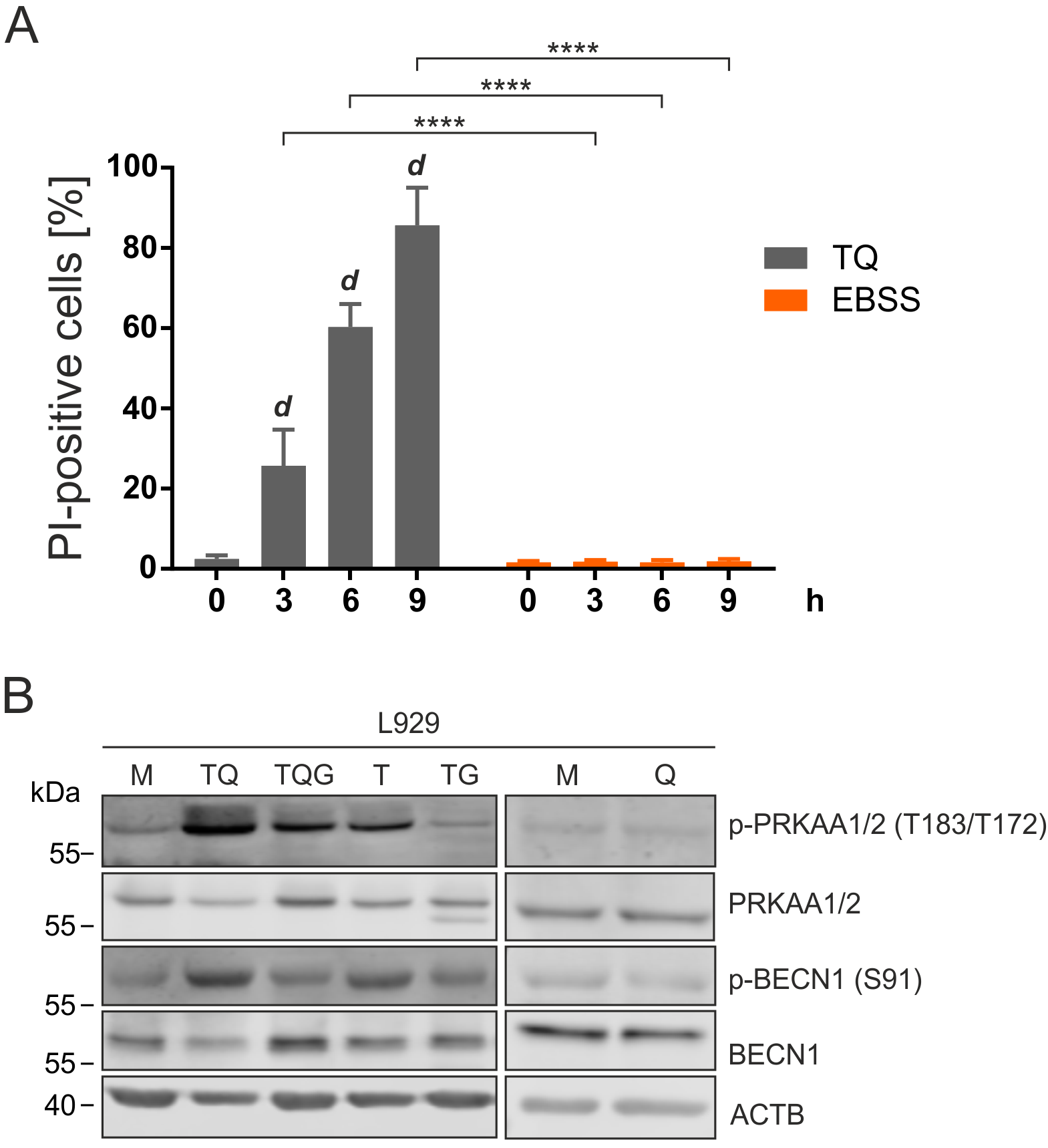
**

**Figure S1.** TNF induces cell death and activates AMPK. (**A**) L929 cells were treated with 10 ng/ml TNF + 30 µM QVD (TQ) or were incubated in starvation medium (EBSS) for the indicated times. Cells were collected and incubated with propidium iodide (PI) for 1 h at 4°C. Then the signal was measured by flow cytometry. Data represent mean + SD. Statistical analysis was performed using repeated measures two-way ANOVA (corrected by Sidak’s multiple comparisons test between treatments and by Tukey’s multiple comparisons test between time points). Statistically significant differences within TQ-treated cells (compared to 0 h TQ) are depicted as letters directly above the bars. **** or *d*: *P* < 0.0001. (**B**) L929 cells were exposed to indicated treatments (medium [M], 10 ng/ml TNF [T], 30 µM QVD [Q], 5 µM GSK'872 [G]) for 6 h. Cells were lysed and cleared cellular lysates were subjected to SDS-PAGE and analyzed by immunoblotting for the indicated proteins.


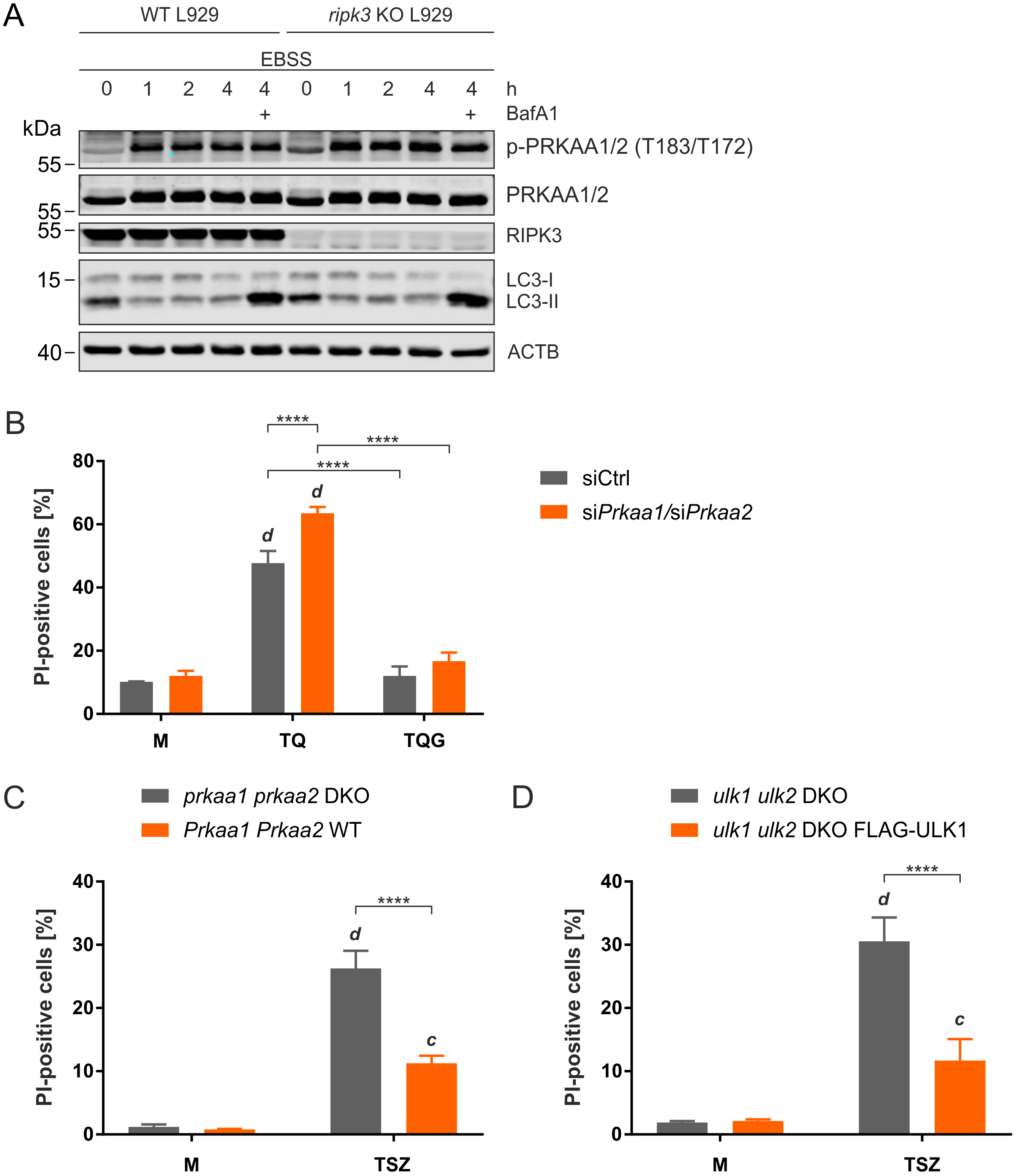


**Figure S2.** RIPK3 is not required for starvation-induced autophagy, but AMPK and ULK1 negatively regulate TNF-induced necroptosis. (**A**) *Ripk3* WT and KO L929 cells were incubated in EBSS with or without 20 nM bafilomycin A_1_ (BafA1) for the indicated times. Then, cells were lysed and cleared cellular lysates were subjected to SDS-PAGE and analyzed by immunoblotting for the indicated proteins. (**B**) L929 cells were transfected with non-targeting (siCtrl) or *Prkaa1/2* siRNAs (si*Prkaa1/2*). 48 h post transfection, cells were left untreated (medium, M) or exposed to 10 ng/ml TNF + 30 µM QVD (TQ) with or without 5 µM GSK'872 (G) for 4 h. Cells were collected and incubated with propidium iodide (PI) for 1 h at 4°C. Then the signal was measured by flow cytometry. Data represent mean + SD from three independent experiments. (**C** and **D**) *Prkaa1/2* WT and *prkaa1/2* DKO MEFs (**C**), or *ulk1/2* DKO MEFs and *ulk1/2* DKO MEFs reconstituted with *Ulk1* cDNA (**D**) were left untreated (medium, M) or treated with 30 ng/ml TNF + 100 nM SMAC-mimetic + 20 µM z-VAD (TSZ) for 5 h. Cells were collected and incubated with propidium iodide (PI) for 1 h at 4°C. Then the signal was measured by flow cytometry. Data represent mean + SD from at least three independent experiments. (**B-D**) Statistical analysis was performed using ordinary two-way ANOVA (corrected by Tukey’s multiple comparisons test). For **B**, statistically significant differences are only indicated for TQ (siCtrl vs. si*Prkaa1/2*) and for TQ vs. TQG (within each siRNA experiment). For **C** and **D**, statistically significant differences are only indicated for TSZ (DKO vs. WT cells). Statistically significant differences to control (medium, M) are depicted as letters directly above the bars. *** or *c*: *P* < 0.001, **** or *d*: *P* < 0.0001.

**
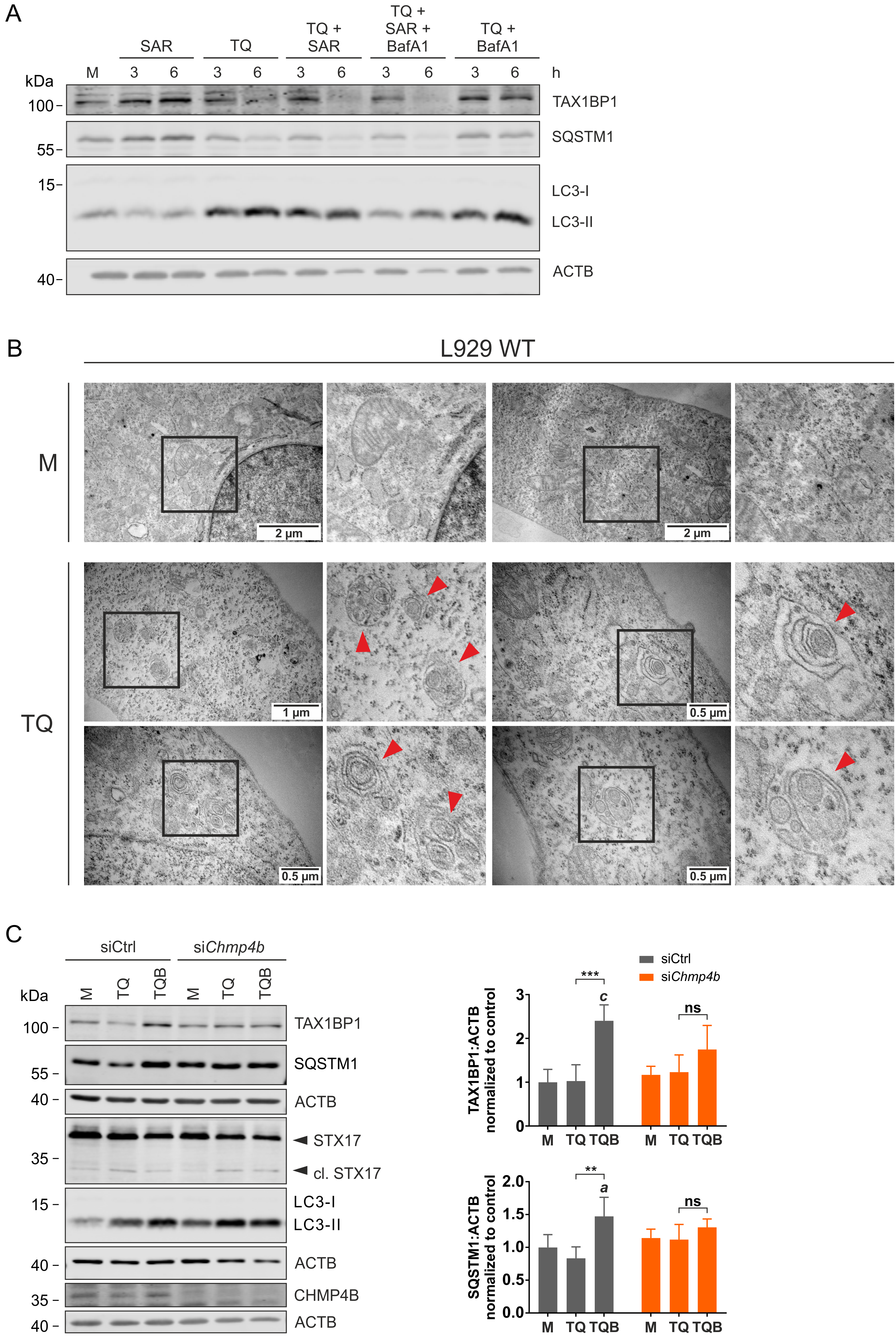
**

**Figure S3.** Inhibition of early autophagy events does not prevent TNF-induced degradation of selective autophagy receptors. (**A**) L929 cells were exposed to indicated treatments (medium [M], 10 ng/ml TNF [T], 30 µM QVD [Q], 5 µM SAR405 [SAR], 20 nM bafilomycin A_1_ [BafA1]) for indicated times. Cells were lysed and cleared cellular lysates were subjected to SDS-PAGE and analyzed by immunoblotting for indicated proteins. (**B**) L929 cells were left untreated (medium, M) or treated with 10 ng/ml TNF + 30 µM QVD (TQ) for 3 h, then cells were fixed and processed according to the protocol described in the Experimental Procedures section. Images were captured by transmission electron microscopy (TEM). Red arrowheads represent multivesicular bodies (MVBs). (**C**) L929 cells were transfected with non-targeting (siCtrl) or *Chmp4b* siRNAs (si*Chmp4b*). 48 h post transfection, cells were left untreated (medium, M) or exposed to 10 ng/ml TNF + 30 µM QVD with or without 20 µM bafilomycin A_1_ (TQ or TQB) for 4 h. Then, whole cell lysates were subjected to SDS-PAGE and immunoblotting for indicated proteins. The density of each protein band was divided by the average of the density of all bands from the same protein on the membrane. Fold changes were calculated by dividing each normalized ratio (protein to loading control) by the average of the ratios of the control lane (siCtrl, medium). Statistical graphics represents mean + SD (n = 4). Statistical analysis was done by ordinary two-way ANOVA (corrected by Tukey’s multiple comparisons test). Statistically significant differences are only indicated for TQ vs. TQB (within each siRNA experiment). Statistically significant differences to control (medium, M) are depicted as letters directly above the bars. * or *a*: *P* < 0.05, ** or *b*: *P* < 0.01*** or *c*: *P* < 0.001, ns: non-significant.

**
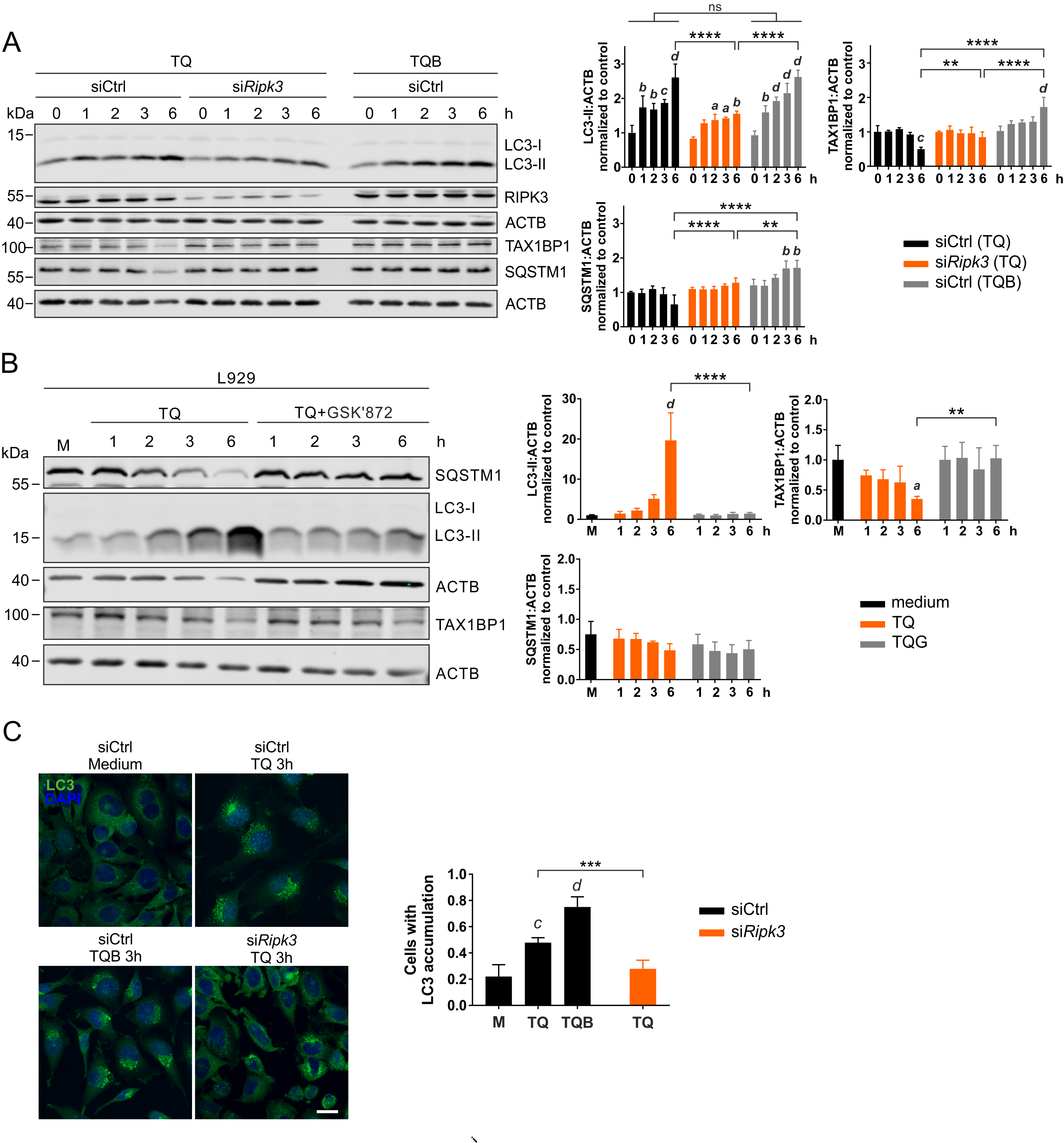
**

**Figure S4.** Necroptosis induced by TNF inhibits LC3 degradation. (**A**) L929 cells were transfected with non-targeting (siCtrl) or *Ripk3* siRNAs (si*Ripk3*). 48 h post transfection, cells were exposed to 10 ng/ml TNF + 30 µM QVD with or without 20 nM bafilomycin A_1_ (TQ or TQB) for indicated times. Then, cells were lysed and cleared cellular lysates were subjected to SDS-PAGE and immunoblotting for LC3, RIPK3, TAX1BP1, SQSTM1/p62 and ACTB. The density of each protein band was divided by the average of the density of all bands from the same protein on the membrane. Fold changes were calculated by dividing each normalized ratio (protein to loading control) by the average of the ratios of the control lane (siCtrl, 0 h TQ). Results are mean + SD (n = 3). (**B**) L929 WT were left untreated (medium, M) or exposed to 10 ng/ml TNF + 30 µM QVD (TQ) with or without 5 µM GSK'872 for indicated times. Then, cells were lysed and cleared cellular lysates were subjected to SDS-PAGE and immunoblotting for indicated proteins. The density of each protein band was divided by the average of the density of all bands from the same protein on the membrane. Fold changes were calculated by dividing each normalized ratio (protein to loading control) by the average of the ratios of the control lane (medium, M). Statistical graphics represents mean + SD (n = 3). (**C**) L929 cells were transfected with non-targeting (siCtrl) or *Ripk3* siRNAs (si*Ripk3*). 48 h post transfection, cells were exposed to indicated treatments (medium [M], 10 ng/ml TNF [T], 30 µM QVD [Q], 20 nM bafilomycin A_1_ [B]) for 3 h. After that, cells were fixed and subjected to LC3 immunostaining using anti-LC3 antibodies (MBL International; PM036) and Alexa Fluor 488-conjugated goat anti-rabbit secondary antibodies. Scale bar: 20 µm. (**A-C**) Statistical analysis was done by repeated measures two-way ANOVA (corrected by Tukey’s multiple comparisons test) for **A** and **B**, by repeated measures two-way ANOVA (corrected by Sidak’s multiple comparisons test) for **B**, by ordinary one-way ANOVA (corrected by Tukey’s multiple comparisons test) for **C**, or by unpaired *t* test with Welch’s correction for TQ comparison in **C**. For **A**, statistically significant differences are only indicated for 6 h treatments (LC3, TAX1BP1 and SQSTM1/p62) and for the comparison TQ vs. TQB (siCtrl, LC3). Statistically significant differences to control (0 h) are depicted as letters directly above the bars. * or *a*: *P* < 0.05, ** or *b*: *P* < 0.01, *** or *c*: *P* < 0.001, **** or *d*: *P* < 0.0001.

**
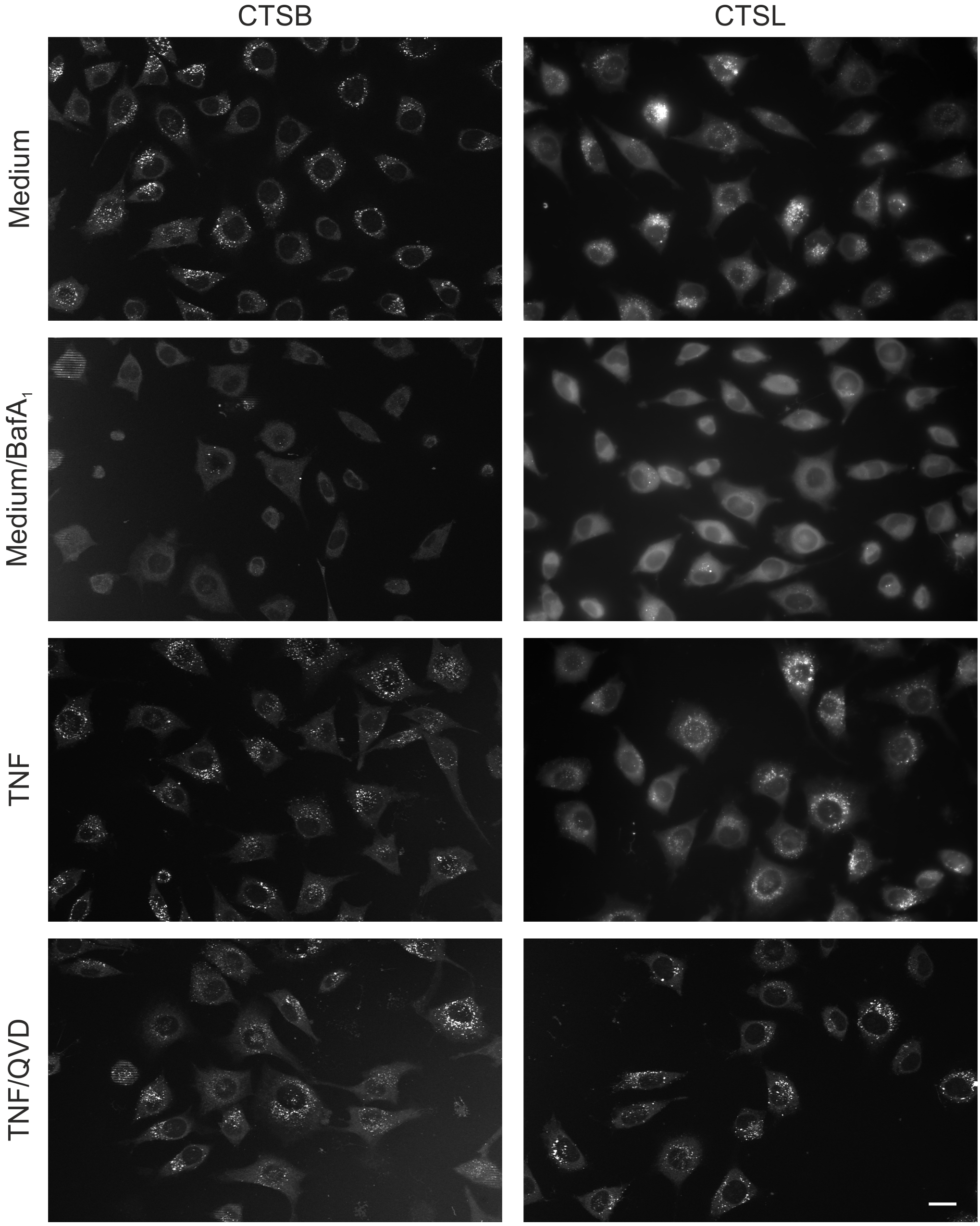
**

**Figure S5.** Necroptosis induced by TNF does not affect activities of CTSB and CTSL. L929 cells were seeded onto 8-well chambered coverglasses and cultured at 37°C overnight. Next day, cells were exposed to indicated treatments (10 ng/ml TNF, 30 µM QVD, 20 nM bafilomycin A_1_, [BafA1]) for 3 h. Then, cells were cultured with the magic red bound substrates of CTSB or CTSL for 15 min at 37°C. After washing 5 times with medium preheated to 37°C, cells were analyzed by fluorescence microscopy. Scale bar: 20 µm.


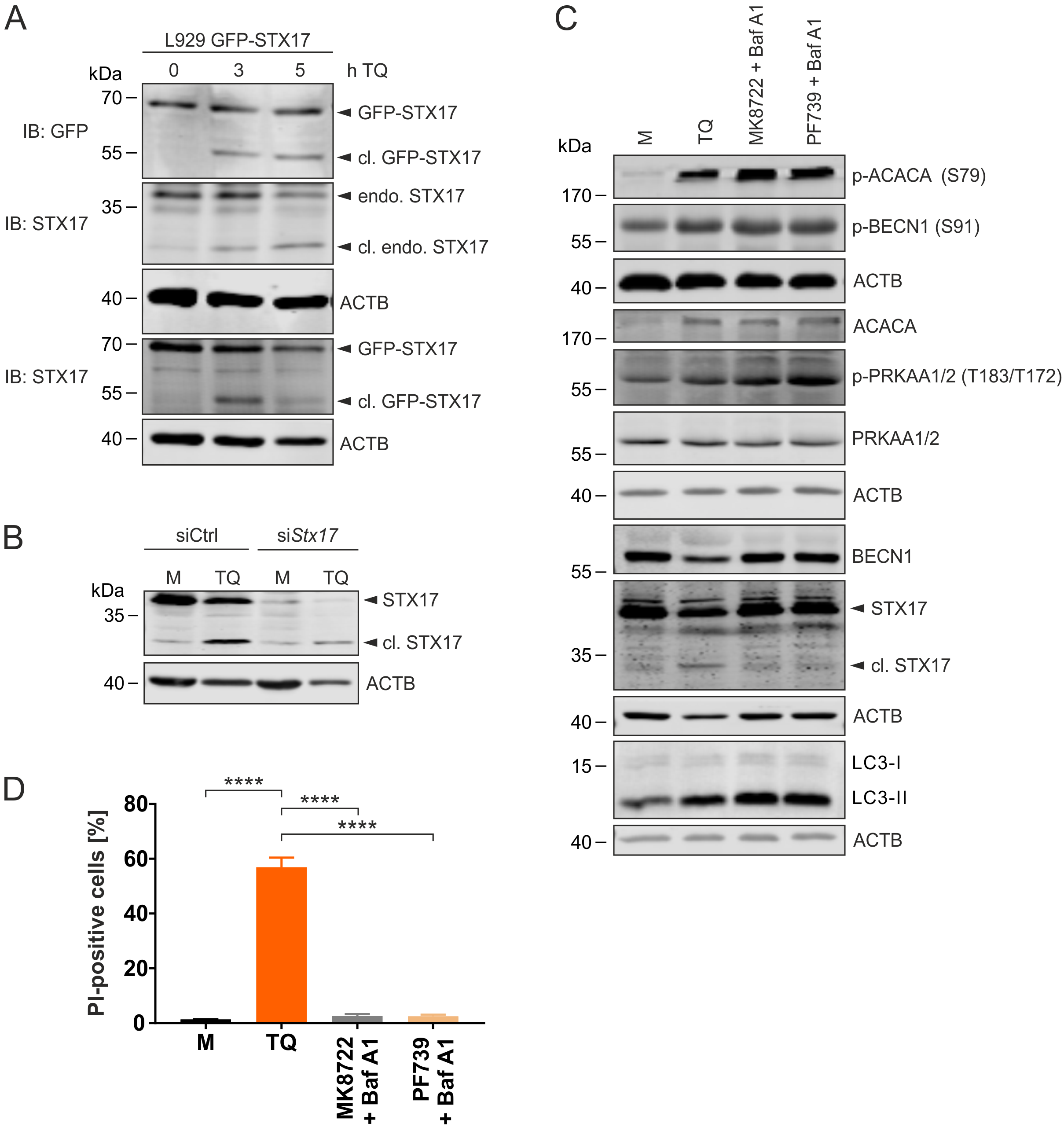


**Figure S6.** TNF/QVD treatment induces STX17 cleavage in L929 cells, but AMPK activation alone is not sufficient. (**A**) L929 stably expressing GFP-STX17 cells were treated with 10 ng/ml TNF + 30 µM QVD (TQ) for indicated times. Then, cells were lysed and cleared cellular lysates were subjected to SDS-PAGE and immunoblotting for indicated proteins. (**B**) L929 cells were transfected with non-targeting (siCtrl) or *Stx17* siRNAs (si*Stx17*). 48 h post transfection, cells were left untreated (medium, M) or exposed to 10 ng/ml TNF + 30 µM QVD (TQ) for 4 h. Then, cells were lysed and cleared cellular lysates were subjected to SDS-PAGE and analyzed by immunoblotting for indicated proteins. (**C**) L929 cells were exposed to indicated treatments (medium [M], 10 ng/ml TNF [T], 30 µM QVD [Q], 5 µM MK8722, 5 µM PF739, 20 nM bafilomycin A_1_ [Baf A1]) for 3 h. Then, cells were lysed and cleared cellular lysates were subjected to SDS-PAGE and immunoblotting for indicated proteins. (**D**) L929 cells were exposed to indicated treatments (medium [M], [10 ng/ml TNF [T], 30 µM QVD [Q], 5 µM MK8722, 5 µM PF739, 20 nM bafilomycin A_1_ [Baf A1]) for 4 h. Cells were collected and incubated with propidium iodide (PI) for 1 h at 4°C. Then the signal was measured by flow cytometry. Data represent mean + SD from three independent experiments. Statistical analysis was performed using ordinary one-way ANOVA (corrected by Tukey’s multiple comparisons test). **** P < 0.0001.
